# Functional Calmodulin States Are Selected from an Electrostatically Tuned Free Energy Landscape

**DOI:** 10.1021/acs.jcim.6c00532

**Published:** 2026-05-16

**Authors:** Busra Tayhan, Sila Horozoglu, Ali Rana Atilgan, Canan Atilgan

**Affiliations:** † Faculty of Engineering and Natural Sciences, 52991 Sabanci University, Tuzla, Istanbul 34956, Türkiye

## Abstract

Calmodulin (CaM) is a versatile calcium-binding protein whose structural flexibility enables regulation of diverse cellular processes, but capturing its full conformational landscape remains challenging due to high energy barriers between states. Here we employ well-tempered metadynamics simulations using physically interpretable collective variables to explore CaM conformations under calcium-bound and calcium-free states at physiological and low salt concentrations. We identify four principal conformations that shift in population depending on calcium binding and ionic strength. Calcium binding favors compact states, while low salt conditions flatten the energy landscape, facilitating transitions, but also causing kinetic trapping due to salt-bridge interactions. Comparison with experimental CaM–protein complexes shows that target binding stabilizes extended conformations distinct from the minima accessible to free CaM, illuminating how calcium and the ionic environment orchestrate CaM’s conformational dynamics in cellular signaling.

## Graphical abstract

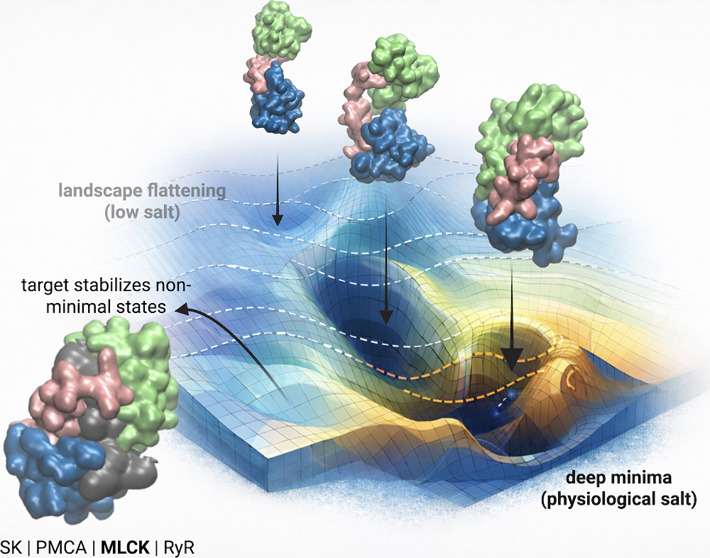

## Introduction

Calcium (Ca^2+^), a ubiquitous secondary messenger,^1^ plays a crucial role in regulating cellular processes such as muscle contraction, exocytosis, gene expression, apoptosis, and metabolism. Its homeostasis is maintained by the coordinated exchange between cellular compartments^2^ which balance storage, signaling, and energy production. Organelles such as the endoplasmic reticulum,^3,4^ Golgi apparatus,^5^ mitochondria,^4,6^ and the plasma membrane^1^ coordinate its movement to maintain homeostasis and support essential cellular functions. Despite its necessity for cellular function, excessive calcium accumulation can be toxic.^7^ To prevent toxicity and maintain Ca^2+^ signaling, the concentration of this divalent ion is tightly regulated by calcium-binding proteins.

Although many proteins interact with Ca^2+^, only a few show high-affinity and specific binding.^8^ Among them, calmodulin (CaM) stands out for its conformational flexibility and functional versatility.^9^ CaM is a small, acidic, and highly conserved Ca^2+^-binding protein present in all eukaryotes.^10^ It has 148 residues and contains two globular domains connected by a linker (Figure 1A). The N-terminal and C-terminal domains span residues 1–68 and 92–148, respectively, each capable of binding two Ca^2+^ ions through EF-hand motifs.^11,12^ The negatively charged residues within these motifs create strong affinity for Ca^2+^ whose binding stabilizes the protein while triggering conformational changes.^13^ The flexible central helix formed by residues 69–91 connecting the two domains imparts controlled flexibility to the protein^11^ which adopts a wide range of conformations.^14^ This flexibility allows CaM to adjust its structure depending on its binding partners.^15^ Ca^2+^ binding reorganizes the domains,^16,17^ either bringing them closer together or moving them apart, directly shaping interactions with target proteins.^13,18^ Although many interactions depend on Ca^2+^, CaM can also bind certain targets in its calcium-free form, reflecting its structural versatility.^19,20^ It can adopt a range of conformations from extended to compact^21^ and engage with binding partners either through both domains or through a single lobe.^22^ This adaptability makes CaM a central regulator of calcium signaling and a mediator of diverse cellular processes.^22^

**Figure 1:**
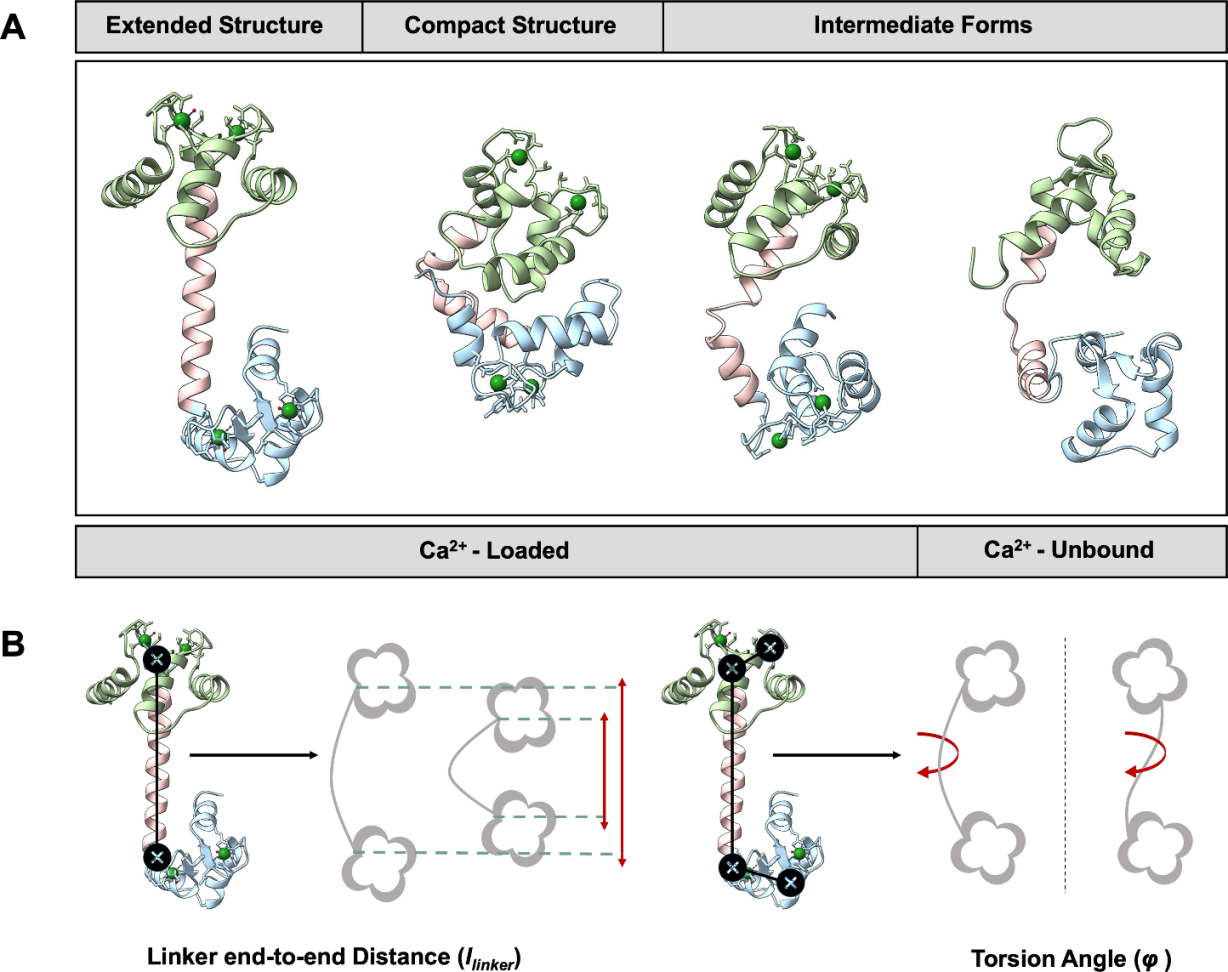
(A) Representative three-dimensional structures of CaM. Ca^2+^-loaded conformations include the extended (PDB ID: 3CLN) and compact (PDB ID: 1PRW) X-ray structures. Intermediate forms are captured in the ensembles of NMR structures, e.g. by the Ca^2+^-loaded CaM (PDB ID: 2K0E) and by the calcium-free CaM (PDB ID: 6Y95); two such intermediate conformers from the respective NMR structures are exemplified here. Ca^2+^ ions shown in green; EF-hand motif residue side chains in licorice representation. (B) Schematization of the CVs utilized in this study.

The distinct conformations of CaM have been extensively characterized through NMR and X-ray crystallography in calcium-bound, target-bound, and unbound forms, as well as fluorescence resonance energy transfer (FRET)^23^ and mass spectrometry (MS).^24^ Some representative structures are shown in Figure 1A.

In its calcium-loaded, peptide-free state, CaM exhibits both extended and compact conformations. X-ray structures often capture an extended dumbbell-shaped arrangement, where the two domains are connected by an elongated helix exemplified by the 3CLN coded Protein Data Bank (PDB)^25^ structure where the two lobes face opposite sides of the linker in a *trans* arrangement.^11^ On the other hand, alternative crystal structures reveal a more compact form with the lobes positioned closer together, e.g. captured in the 1PRW coded PDB structure where the lobes face the same side of the linker in a *cis* arrangement.^26^ In the extended state, CaM exposes hydrophobic pockets that mediate interactions with calcium-regulated enzymes such as kinases and phosphatases, as well as ion channels including the ryanodine receptor and voltage-gated calcium channels, which are central to calcium homeostasis and excitability.^13,27,28^ Ca^2+^-bound, ligand free NMR structures further emphasize CaM’s intrinsic flexibility, displaying a continuum from extended to compact states (e.g., the 160 NMR ensemble structures in PDB ID: 2K0E).^10^ Calcium binding alone does not confine CaM to a single structure but primes it for dynamic transitions.^10^ When bound to target peptides, such as myosin light chain kinase (MLCK), CaM adopts a compact conformation, wrapping tightly around hydrophobic motifs to mediate specific regulatory interactions.^16,17,28^ In the absence of calcium, *apo* CaM typically adopts an intermediate conformation with partially collapsed lobes that retain flexibility;^15^ these are exemplified in the 30 NMR structures of PBD ID: 6Y95.^29^ This state is expected to prime CaM for recognition of conserved IQ motifs in voltage-gated sodium channels and unconventional myosins,^20^ providing calcium-independent regulation of neuronal signaling and intracellular trafficking.^28^

Experimental evidence from FRET and MS studies further supports the conformational flexibility of CaM. Both techniques indicate that calcium-loaded CaM predominantly adopts a compact conformation, challenging the traditionally assumed dumbbell-shaped structure.^23,24^ MS data also show that this globular shape of CaM allows for stable peptide binding, with interactions occurring across both lobes.^24^ Additionally, FRET experiments reveal that *apo* CaM is more extended compared to the globular Ca^2+^-loaded state and supports the view that it exhibits an intermediate conformation.^23^

Despite extensive experimental and computational studies, fully capturing the conformational landscape of CaM remains a challenge. Experimental techniques such as NMR and X-ray crystallography provide representations of CaM’s different states but fail to reveal the numerous transitions between them. Molecular dynamics (MD) simulations offer dynamic insights into the effect of environment on protein conformation and dynamics for this and other metal binding proteins.^30–32^ However, these are often restricted by limited time scales of the simulations, which are insufficient to sample the rare transitions between distinct conformational states.^33,34^ Given the importance of conformational multiplicity in CaM’s ability to regulate cellular processes, applying enhanced sampling techniques^35–38^ might play a crucial role in bridging this gap.

In this study we employed well-tempered metadynamics (MetaD),^39^ a powerful enhanced sampling method that allows us to efficiently explore the free energy landscape of CaM. MetaD introduces a bias potential along selected collective variables (CVs), allowing the system to escape local minima via iterative deposition of Gaussian hills. Once the CVs are sampled with approximately uniform probability, the simulation is halted and the negative of the accumulated bias reveals the underlying free-energy surface. Well-tempered MetaD refines this approach by gradually reducing bias deposition, preventing overfilling and yielding a more accurate reconstruction of the thermodynamic landscape.^39^ Since convergence and a realistic projection of the true free energy surface strongly depends on the choice of the CVs,^40^ their poor selection can cause inadequate sampling and obscure key conformational changes.^41^

Different types of CVs may be selected depending on how effectively they represent the motions of the system. They may be selected from physics-based reaction coordinates, such as interatomic distances, angles, dihedrals, or the radius of gyration of a subset of atoms in the system. Alternatively, CVs can be deduced from MD trajectories, such as principal components (PCs) or time-lagged independent components (TICs).^42^ The advantage of selecting physics-based reaction coordinates is that, when comparing the same system under slightly different conditions, the changes may always be followed intuitively and comparatively.^43^ Conversely, when using PCs or TICs, there is no guarantee that the projections reflect similar phenomena which renders interpreting shifts in the energy landscape tricky.^43,44^ The use of machine-learning-derived collective variables, such as those obtained from time-lagged autoencoders^45^ or VAMP-based approaches,^46^ may also help identify nonlinear CVs directly from simulation data. In the present system, such data-driven CVs could help capture couplings among linker bending, interlobe reorientation, and salt-bridge rearrangement that may not be fully resolved by the current two-dimensional description. However, the present physics-based CVs remain advantageous for direct mechanistic interpretation and for systematic comparison across calcium-loading and ionic-strength conditions. Therefore, we used two physics-based reaction coordinates that were shown to capture the motions of CaM: the rotational motion of its lobes relative to each other and the change in their separation (Figure 1B).^30^ These DoFs were defined to project MD trajectories into conformational subspaces to provide a detailed understanding of population shifts among CaM’s conformational states and have also been applied in other contexts.^47^

We leverage well-tempered MetaD in combination with classical MD simulations to map how calcium loading, ionic strength, and the ensuing electrostatic environment shape the conformational free-energy landscape of CaM. Mapping conformations on the landscape captured by our predefined CVs allows us to capture rare transitions, identify previously uncharacterized minima, and distinguish thermodynamically favored states from kinetically persistent ones. By integrating these computational landscapes with experimentally determined CaM–target complexes, we show that CaM’s functional conformations are selected from a tunable electrostatic landscape rather than predetermined by its intrinsic minima. This framework provides a mechanistic basis for how CaM adapts its structure to cellular context and target engagement, offering broader insights into how electrostatics and environmental cues regulate protein conformational ensembles.

## Methods

### System Preparation

To investigate how the absence or presence of Ca^2+^ ions and changes in salt concentration influence CaM’s conformations, we performed classical MD (cMD) and well-tempered MetaD simulations (labeled simply MetaD in the rest of the manuscript) under each condition (Table 1). The calcium-loaded, extended structure of CaM serves as the canonical form of CaM in the literature; thus, we used the 3CLN structure (Figure 1) as the starting point for system preparation. We labeled the systems as H^P^ (calcium-loaded *holo* CaM at physiological salt concentration), H^L^ (*holo* CaM in low salt), A^P^ (Ca^2+^-free *apo* CaM at physiological salt concentration) and A^L^ (*apo* CaM at low salt), respectively. To simulate *apo* conditions in these initial systems, Ca^2+^ ions were removed from the 3CLN structure. By using the solvate plug-in of VMD,^48^ we solvated the protein structures in a rectangular water box with a minimum distance of 10 Å between the protein and the nearest edge. We added K^+^ ions to neutralize charges in low salt simulations, with additional K^+^ and Cl^–^ ions to mimic physiological ion concentration (see Table 1).

**Table 1:** Summary of Systems Simulated

System label^a^	Equilibrated box size (Å^3^)	Salt ions (K^+^/Cl^–^)	Ionic strength (mM)	Simulation time (cMD/MetaD/cMD)
H^P^	65.3 × 82.4 × 61.8	43/28	177	2 × 1 μs/2 × 800 ns/4 × 0.2 μs
A^P^	65.1 × 82.3 × 61.6	51/28	199	2 × 1 μs/2 × 800 ns/4 × 0.2 μs
H^L^	65.3 × 82.4 × 61.7	15/0	38	2 × 1 μs/2 × 800 ns/4 × 0.2 μs
A^L^	65.3 × 82.5 × 61.8	23/0	57	2 × 1 μs/2 × 800 ns/4 × 0.2 μs

a H/A labels indicate *holo*/*apo* CaM; superscripts P/L indicate physiological/low salt conditions.

### cMD Simulations

We performed duplicate 1 μs long cMD simulations under each condition by using the NAMD software package,^49^ with the CHARMM36 force-field to model the protein and the TIP3P model for water molecules.^50^ We utilized VMD for preprocessing of structures, such as protein structure file (PSF) generation, solvation (constructing water-box), ionization and visualization of MD trajectories.^48^ We employed particle mesh Ewald method for calculating long-range electrostatic interactions with a cutoff of 12 Å with a switching distance of 10 Å. We applied the RATTLE algorithm to constrain bonds, enabling a 2 fs time step with Verlet integration. We used Langevin piston for pressure control at 1 atm, and we controlled the temperature at 310 K by the Langevin thermostat. We minimized each system for 10000 steps, and performed equilibrium simulations in the *NPT* ensemble. We stored the trajectories every 100 ps.

We used the first frame of each trajectory as a reference for root-mean-square deviation (RMSD) calculations. We performed radial distribution function, *g*(*r*), calculations in VMD with the periodic boundary conditions taken into account and the distances binned in 0.1 Å increments.^48^ For these calculations, we selected K^+^ and Cl^–^ ions as the first reference group components, and the side chains of negatively charged residues (GLU or ASP) as the second reference group. We carried out trajectory analyses and RMSD calculations with the ProDy package^51^ within the Python programming environment. We made all in-house scripts used in this study available on GitHub.

### Well-Tempered Metadynamics (MetaD) Simulations

Two geometry-related CVs were defined to efficiently explore the conformational landscape of CaM (Figure 1B). To represent the switch between extended and compact conformations of CaM, following the methodology we introduced in prior studies,^30^ one CV is the linker end-to-end distance (*l*_linker_) defining the distance between the C_α_ atoms of residues 69 and 91 marking the end points of the linker region. The second CV is the coarse-grained torsion angle (φ) describing the positioning of the two lobes relative to each other. This torsion angle is defined using, in order, the center of mass of the N-terminal domain, the C_α_ atoms at the beginning and end of the helical linker region, and the center of mass of the C-terminal domain.

To initiate the metadynamics simulations, we selected one representative snapshot for each condition from the cMD trajectories. The selection was guided jointly by the RMSD profiles and the distribution of conformations in the (*l*_linker_ *,φ*) space. We identified snapshots belonging to the minima sampled in the (*l*_linker_,φ) projections (Figure S2) and chose frames from time intervals where the RMSD exhibited only small fluctuations (Figure S1), indicating local structural stability. Thus, the starting conformers used for MetaD represent stable basins sampled during the unbiased cMD simulations.

Because these snapshots were extracted from ongoing MD trajectories, they retained solvent configurations, ion distributions, and atomic velocities specific to the instantaneous cMD frame. To ensure a consistent starting protocol for all MetaD simulations and to avoid bias from a particular solvent/ion arrangement, we removed water molecules and ions, rebuilt the solvent box, and added ions to reproduce the intended salt condition for each system, following the same preparation procedure used for the cMD simulations.

We minimized each system for 10000 steps, followed by a 100 ns equilibrium simulation to reach a stable local minimum. This approach ensured that the starting configurations for the MetaD simulations began at well-defined minima, allowing Gaussian hills to be deposited effectively for enhanced sampling. We then started MetaD simulations using the final atomic coordinates and velocities. We defined the upper and lower limits of *l*_linker_ as 10–50 Å, divided into 1.5 Å grid intervals, while we divided φ into 10° grids spanning the range [-π,π]. The height and width of the Gaussian were 0.2 kcal mol^–1^ and 1.0 Å respectively. The bias factor for well-tempering was set to 6, and hills were deposited every 500 steps. The bias factor was selected based on commonly used values in the WT-MetaD literature. We then verified it empirically for satisfactory sampling and stable PMF reconstruction for the present system. For each system, two independent MetaD simulations were carried out for 800 ns each.

For PMF analysis, the duplicate trajectories for each condition were combined. Distinct conformational basins were identified by inspection of the PMFs for the corresponding condition. To estimate free-energy differences between states, we considered the last 600 ns of each MetaD replica, divided this portion into three 200 ns blocks, and calculated the occupation probability of each identified basin within each block. Basin free energies were then obtained by Boltzmann reconstruction from these probabilities. The reported free-energy differences correspond to the mean over the six 200 ns blocks obtained from the two duplicate trajectories, and the uncertainty is given as the standard error of the mean. In addition, to provide a measure of basin depth, we also report the minimum PMF value attained within each basin.

### cMD Simulations of Four MetaD Sampled Conformers under Different Conditions

We selected four conformers representing the compact/extended structures where the two lobes face the same side (*cis* arrangement) or opposite sides (*trans* arrangement) of the linker from the MetaD simulations for further tests of their stability under the prevailing conditions. Selection and labeling of these structures are detailed in the Results and Discussion section. We subjected each of these structures to additional MD simulations under the conditions of H^P^, A^P^, H^L^ and A^L^. After selecting the conformers, we removed water molecules and salts from these snapshots. We carried out 10000-step minimization followed by 200 ns run for each structure, using the same MD simulation protocol as the original cMD simulations.

## Results and Discussion

To dissect how calcium loading and ionic strength shape the conformational landscape of calmodulin, we first examined the extent to which unbiased MD can sample the relevant structural states under each condition with duplicate 1 μs runs (see RMSD profiles in Figure S1). As expected, cMD simulations were not efficient in sampling possible *apo*/*holo* conformations of CaM under the different environmental conditions. In all but one case, the linker maintained its original rigid α-helical structure (*l*_linker_ in the range of 30–35 Å), while in all cases the two lobes displayed a repositioning with respect to the initial 3CLN structure traced by the torsional CV, φ, from an initial *trans* positioning of 100° to a predominant sampling in the range (−180,0°) (Figure S2). The highest mobility is observed in the H^L^ system, and from the RMSD plots of the individual cMD runs, we can see that one of the two trajectories is responsible for the jump to the compact conformer at ca. 400 ns time point (Figure S1). Focusing on the RMSD of individual domains, we find that the N-lobe is always more mobile than the C-lobe irrespective of the environmental conditions, more so in the *apo* systems than the *holo* systems. These observations are in agreement with experiments where the fast internal mobility of the N-terminal domain have long been established via NMR experiments,^52^ and its lower calcium affinity has been measured under low salt and physiological conditions.^53^ While cMD simulations concur with the relative flexibilities of the two domains, it is also evident from Figure S2 that the observed conformers are a small subset of those that are physically available to CaM, represented by the larger dots. To sample the full conformational space available to CaM under these differing conditions, we resort to MetaD simulations in what follows.

### Effects of Calcium Loading to CaM Conformers at Physiological Salt

The convergence behavior of the MetaD simulations is shown in Figure S3. The major basins stabilize by approximately 400 ns, indicating that the principal features of the PMFs are established on this time scale. We nevertheless extended each simulation beyond this point to further verify the stability of the reconstructed free-energy landscapes and the relative basin depths. Interestingly, we find the relative positioning of the two lobes, represented by the torsional CV, φ, is sampled on a faster time scale than *l*_linker_. This difference in the time scales is a testament to the difficulty of positioning the two highly negatively charged lobes in proximity and the requirement of finding a facilitating arrangement in the system to reach these compact conformers.

To systematically analyze the free energy landscape, we normalize the results for each system taking the open dumbbell-shaped crystal structure of CaM (3CLN) as a reference. The CVs of the reference structure are calculated and mapped to the PMFs for each system. Energies corresponding to (*l*_linker_, φ) values of the reference structure are extracted and set as the zero of the PMF in each case, serving as baselines for normalization. The PMF plots are then shifted to compare all surfaces with respect to this common reference energy level (Figure S4); the two-dimensional projections of these surfaces are displayed in Figure 2 where the color bar reflects this standardized range, from −9 to 6 kcal/mol. For reference, positions of the experimental structures are also overlaid onto the surfaces of Figure 2 where black dots represent NMR structures determined for *holo* (2K0E) or *apo* CaM (6Y95); and teal dots mark crystal structures (3CLN and 1PRW).

**Figure 2:**
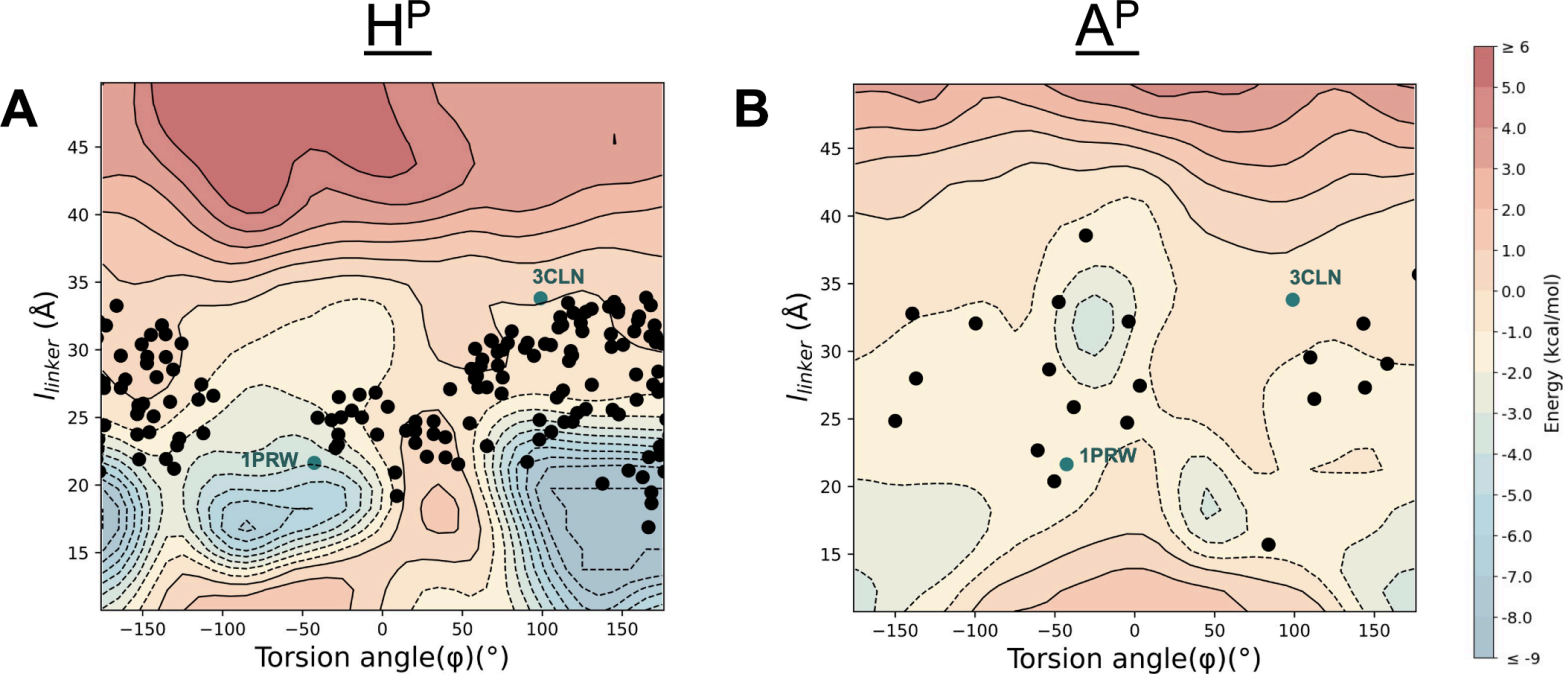
Free energy surface of CaM obtained from MetaD in physiological salt concentrations; (A) *holo* CaM and (B) *apo* CaM. Black dots represent NMR (2K0E for *holo*, 6Y95 for *apo* CaM) structures while teal dots are labeled with the PDB code of the crystal structures they represent.

The PMF of H^P^, obtained from MetaD (Figure 2A) reveals two minima, both attaining a compact form, separated by 1.3 ± 1.2 kcal/mol; however, they are separated by high energy barriers with the minima located at respectively at −17.0 and −15.3 kcal/mol with respect to the 3CLN structure. First minimum is quantified by the wide distribution around φ = 150° and a linker distance of up to 20 Å. We label this conformation where the N- and C-lobes face opposite sides *trans*-compact whose deep minimum is located −17.0 kcal/mol. A second compact minimum is centered at φ = −85.0° and a linker distance of 16.8 Å. Notably, the 1PRW X-ray structure maps onto this second basin. In terms of MetaD sampling, we find that the former is highly stabilized by salt bridges so that if the sampling enters this basin early, it gets stuck therein. In a later subsection, we will discuss the role of these salt bridges in compact conformations which is a recurring observation in many of our trajectories. Compact states are also observed in NMR structures of *holo* CaM (2K0E); some of which even more compact than the 1PRW X-ray structure. Mass spectrometry experiments show that CaM predominantly adopts compact conformations in fully Ca^2+^ loaded CaM,^24^ supporting our MetaD findings. While few of the 160 representative NMR-deduced structures mapped onto the PMF are compact, the majority occupy the regions that are defined by the basin mapped by MetaD. We note that these experimental data were collected^54^ at 100 and 10 mM and collated to generate the 2K0E conformers.^10^ We will discuss the PMF obtained under low salt conditions in the next subsection.

In the A^P^ system (Figure 2B), three minima are observed. The major minimum corresponds to a *cis*-extended conformer in which the two lobes face the same side of the linker, centered around φ = −30° with a linker distance *l*_linker_ > 25 Å. The free energy at the minimum is deep with a value of −7.1 kcal/mol with respect to our reference 3CLN position. Two additional compact minima are located at approximately (φ = 45°, *l*_linker_ = 16 Å; *cis*-compact) and (φ = 175°, *l*_linker_ = 12 Å; *trans*-compact), with minima at −5.8 and −6.2 kcal/mol, respectively. The free energies of these basins are higher than the extended conformer at the major basin, with differences of 1.6 ± 0.5 and 0.3 ± 0.3 kcal/mol, respectively. The structures from the NMR data set of *apo* CaM (PDB ID: 6Y95), all collected at 105 mM, align closely with the extended basin on the PMF. Thus, we conjecture that the *apo* structure under physiological conditions predominantly samples an extended state with a rigid yet rotatable linker, while also accessing less populated compact substates. Such orientational flexibility effectively exposes both EF-hand domains to the surrounding solvent, thereby facilitating efficient sampling of the local Ca^2+^ environment. This intrinsic positional freedom is consistent with CaM’s role as a high-sensitivity Ca^2+^ sensor, whereby it permits both lobes to act semi-independently so that the protein can rapidly engage available ions from multiple spatial directions.

### Effects of Low-Salt Conditions on CaM Conformations

While CaM is not typically found in low-salt conditions, **but rather** in intracellular environments of ∼150 mM salt, there is ample literature where its structure and biochemistry is scrutinized under the former conditions. This is mainly because Ca^2+^ binding and domain–domain contacts in CaM depend heavily on electrostatics. Reducing ionic strength with low-salt buffers minimizes charge screening, amplifying electrostatic effects. This helps reveal details about Ca^2+^ binding cooperativity, interdomain communication, and long-range conformational coupling that are otherwise masked at physiological salt. Moreover, at low ionic strength, fewer ions compete with Ca^2+^ for acidic residues, making the intrinsic binding constants easier to measure. Classic studies used these conditions to separate N- and C-lobe affinities unambiguously^53^ and led to the conclusive result that Ca^2+^ binding is cooperative within each of the domains while there is no indication of cooperativity between the domains.

Notably, local ionic microenvironments, e.g., near negatively charged membranes or within protein complexes, can transiently reduce the effective electrostatic screening experienced by CaM, even when the bulk ionic strength remains physiological. In such contexts, counterion accumulation, co-ion exclusion, and geometrical confinement enhance long-range electrostatic interactions in ways that resemble low-salt conditions *in vitro*. Thus, low-salt experiments can serve as controlled physical probes for uncovering coupling mechanisms and long-range interactions that remain operative, though partially masked, under normal intracellular ionic strengths. To investigate the effect of decreasing monovalent ions in the environment on the conformations of CaM, we performed duplicate 800 ns-long MetaD simulations of *apo* and *holo* CaM mimicking low salt conditions (only K^+^ present in the periodic cell) (see Table 1 under Methods). We then applied the same normalization and scaling procedure as before to systematically analyze their free energy landscape with respect to the position of the canonical 3CLN conformation which is placed at 0 kcal/mol as reference.

Compared to their physiological salt counterparts, the PMF of both H^L^ and A^L^ exhibits shallower minima separated by lower energy barriers (Figure 3). H^L^ maps a large basin on the conformational surface; but we can distinguish two lower energy regions, each corresponding to a specific structural conformation (Figure 3A). The first lies within a wide basin spanning torsion angles in the range [−100°, 100°] and linker distances of 10–38 Å, and includes both compact and extended minima that are predominantly *cis*-oriented, with a minimum at −1.6 kcal/mol. The second distinct region is located near φ ≈ ± 180°, with linker distances restricted to 10–24 Å, labeled *trans*-compact conformation with a minimum at −0.1 kcal/mol. These two basins are separated by an energy difference of 1.0 ± 0.6 kcal/mol.

**Figure 3:**
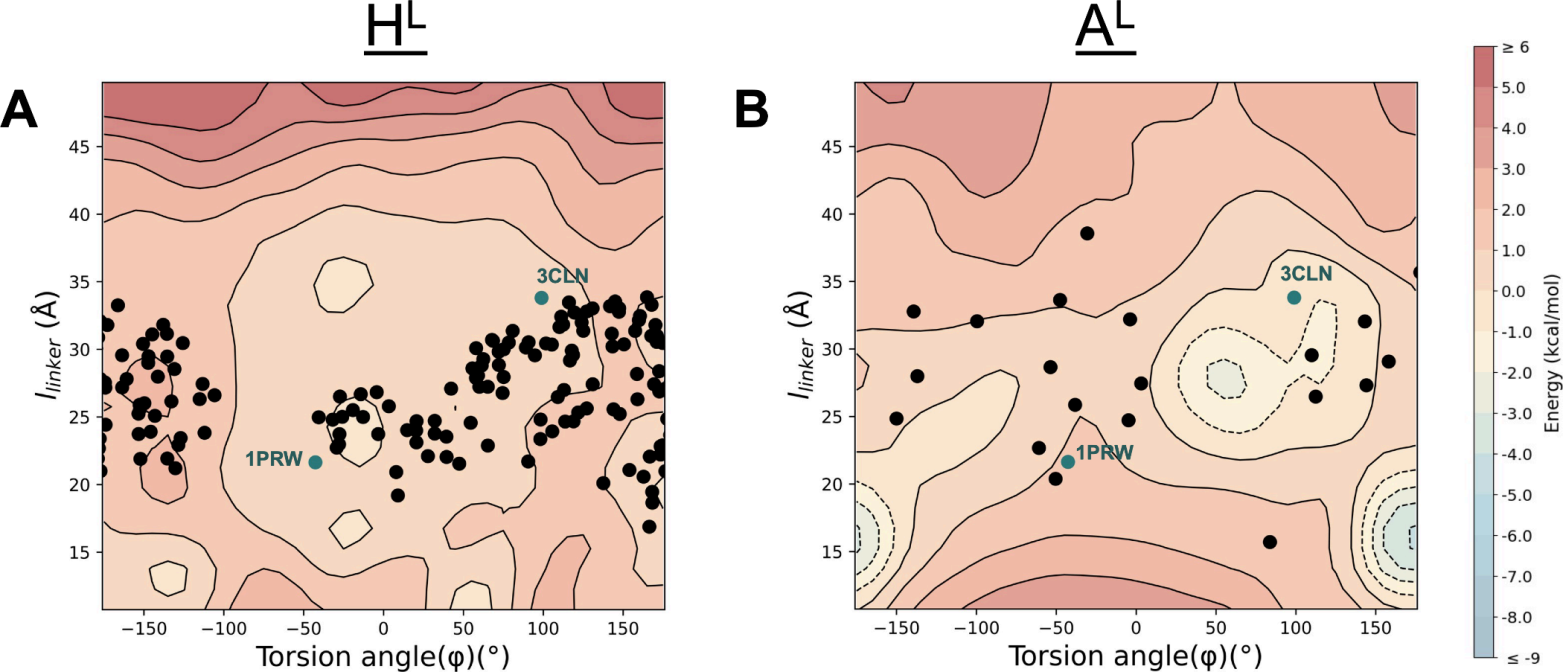
Free energy surface of CaM obtained from MetaD in low salt; (A) *holo* CaM and (B) *apo* CaM. Dots represent the same NMR and crystal structures as in Figure 2.

We also see that the regions of extended *cis* and *trans* conformations are accessible in this ionic strength as relatively low-lying energy regions. Most of the representative NMR conformers deposited under PDB ID 2K0E populate the same regions of the free-energy surface identified in our low-salt simulations. As noted earlier, these experimental structures were derived from data sets acquired at two distinct ionic strengths (10 and 100 mM), preventing unambiguous assignment of individual conformers to a specific condition. Nonetheless, their ensemble distribution is consistent with the conformational basins resolved by our MetaD analysis, indicating that when we consider our H^P^ and H^L^ simulations together, they faithfully capture experimentally accessible conformations.

The A^L^ PMF contains two shallow basins (Figure 3B). The first is characterized by a torsion angle of around 50° and a linker distance centered near 20 Å, corresponding to a more extended conformation, with a minimum at −3.2 kcal/mol. This conformer is consistent with the open, dumbbell-like architecture of *apo* CaM and lies closest to the canonical 3CLN structure (Figure 1). The second minimum is located near φ ≈ ± 180°, with linker distances restricted to 12–20 Å at −5.5 kcal/mol, in a *trans*-compact conformation. The free-energy difference between these two distinct basins is 0.8 ± 0.4 kcal/mol. Both are markedly shallower than those in the physiological-salt systems, reflecting a softer energy landscape for A^L^. It is worth noting that our *apo* simulations span distinct ionic strengths of 199 mM for A^P^ and 57 mM for A^L^, while the experimental *apo* NMR ensemble (PDB 6Y95) was acquired at an intermediate ionic strength of 105 mM. Although this complicates direct comparison, the experimental structures fall within the broader conformational regions predicted by our A^P^ and A^L^ surfaces, supporting the robustness of the simulated landscapes.

These results show that lowering the ionic strength substantially softens the conformational energy landscape of CaM, promoting facile interconversion among compact and extended states in both the *holo* and *apo* forms. For *holo* CaM, reduced ionic screening stabilizes additional compact substates that are separated by only small energetic differences, facilitating rapid interdomain rearrangements. For *apo* CaM, the shallow *trans*-extended minimum reflects an intrinsically flexible, weakly biased landscape that readily accommodates repositioning of the two lobes around an elongated central linker. Such energetic flattening is consistent with a scenario in which reduced ionic strength enhances long-range electrostatic coupling while lowering the penalties associated with domain reorientation. Although CaM typically operates at physiological salt concentrations, local electrostatic microdomains, such as those near membrane surfaces, within crowded assemblies, or in regions of fluctuating ion flux, may transiently reduce effective screening, creating environments that electrostatically approximate low-salt conditions. Our findings therefore suggest that CaM retains the capacity to explore a broadened conformational repertoire within such environments, providing a biophysical basis for its rapid structural adaptability during target engagement and signaling.

### Stability and Kinetic Accessibility of Minima Assessed by Classical MD Simulations

To determine whether the conformational minima identified through MetaD simulations correspond to kinetically stable states or merely reflect transiently sampled basins, we next examine their dynamical behavior using unbiased cMD simulations. While MetaD provides a comprehensive reconstruction of the free-energy landscape, the addition of bias accelerates barrier crossing and may allow sampling of states that are not dynamically stable under equilibrium conditions. cMD therefore offers a complementary view by testing whether these minima persist, interconvert, or dissipate when the system evolves solely under the physical force field. To this end, we extract representative structures from the principal MetaD basins across all systems, subject them to minimization and equilibration, and monitor their spontaneous dynamics projected onto our selected CVs. This approach allows us to evaluate the robustness of the MetaD-derived minima, identify potential kinetic traps, and dissect the role of ionic strength and Ca^2+^ loading in stabilizing or destabilizing distinct conformational states.

We identify four representative conformers from the MetaD simulations each representing the structures sampled under the various conditions (Figure 4A). Going counterclockwise the quadrants of our coarse-grained conformational space, **I** is a *trans*-extended, **II** is a *cis*-extended, **III** is a *cis*-compact and **IV** is a *trans*-compact conformer. Interestingly, while each of these might dominate one of the conditions (**I** ↔ A^L^; **II** ↔ A^P^; **III** ↔ H^L^; **IV** ↔ H^P^, they may still be sampled in more than one condition. In particular, the shallow energy landscape in low salt conditions (Figure 3) is expected to lead to easy passage between the minima.

**Figure 4:**
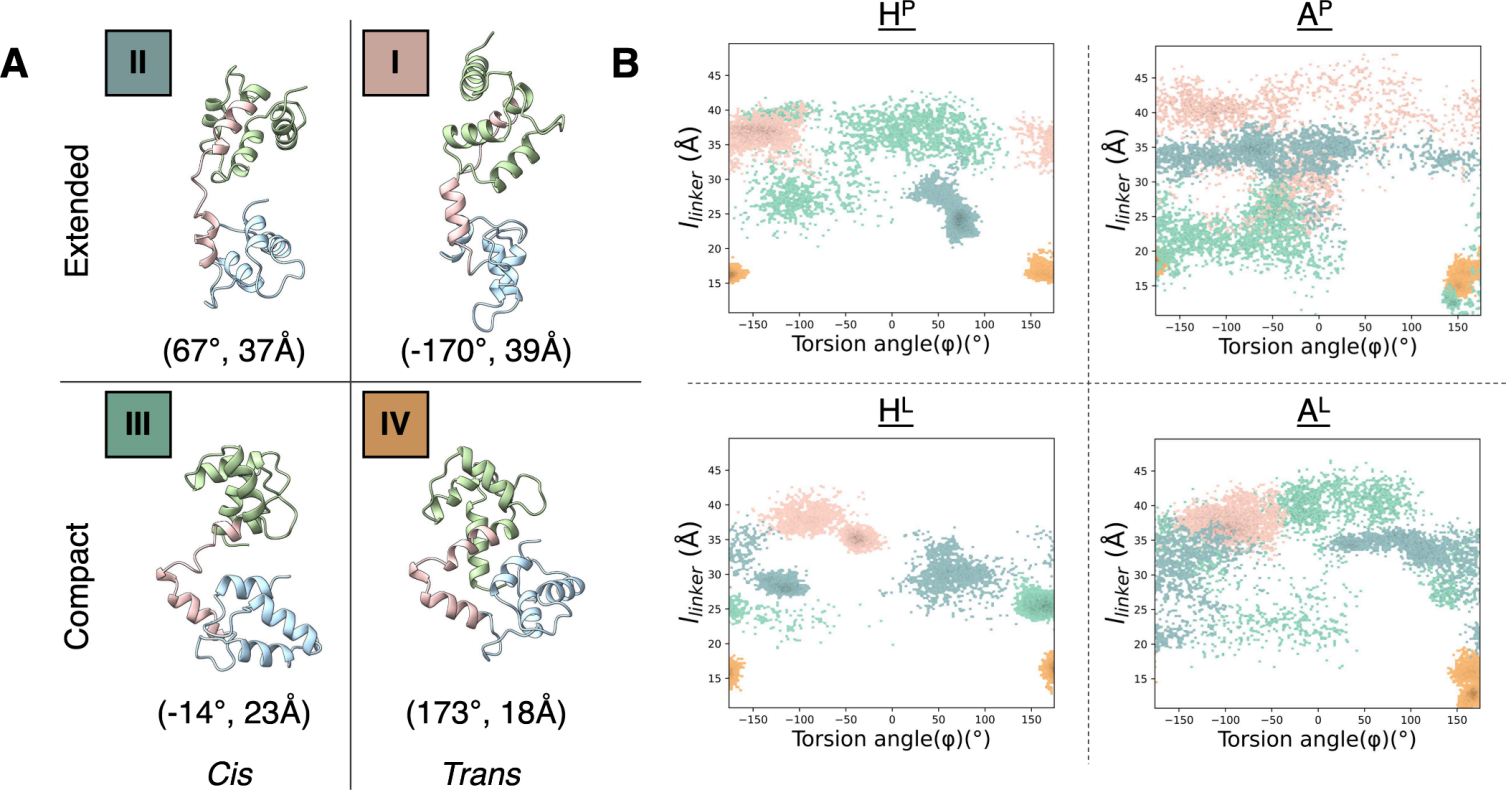
(A) The four conformers selected from the PMFs. Their three-dimensional structures and the specific values of the (φ, *l*_linker_) are displayed. The color labels are **I** = pink, **II** = teal, **III** = mint, and **IV** = ochre. (B) The 4 × 200 ns time points visited under each condition by each conformer are displayed on the CVs with the corresponding colors.

We select snapshots corresponding to these four local minima from our H^L^ MetaD run and then subject them to the appropriate conditions as described under the Methods section to reproduce 200 ns-long cMD simulations for each setup. The coordinates so obtained are projected onto the two-dimensional space in Figure 4B, color coded according to the initial structure I–IV.

We make a couple of general observations from these simulations: First, we find that the *apo* systems display a larger conformational freedom than their corresponding *holo* counterparts in the same salt environment. This is to be expected as *apo* CaM lacks the Ca^2+^-mediated intradomain stabilization that normally rigidifies the EF-hand motifs, thereby weakening interhelical packing and permitting larger-amplitude motions of the two lobes relative to the linker. This observation was not fully recovered in the original 2 × 1 μs cMD simulations which all started from the 3CLN structure (Figure S2). Therein, although we observed this extra flexibility in the overall RMSD profiles in the *apo* forms compared to the *holo* forms in Figure S1, this flexibility did not usually translate into sampling of multiple conformers. Our second general observation is that, in contrast to the other conformers, **IV** exhibits striking kinetic rigidity; it remains trapped near its *trans*-compact conformation in every condition, including those where it is not a thermodynamic minimum.

Beyond these trends, in the H^P^ system, three of the four conformers (**I**, **II**, and **IV**) remain tightly localized during the cMD trajectories, consistent with their positions in stable or moderately stable regions of the H^P^ free-energy landscape (Figure 2). Conformer **IV**, which corresponds to the global *trans*-compact minimum of the H^P^ PMF, is the most confined, but conformers **I** and **II** also remain in relatively well-defined regions. While **II** is drawn to a more compact form within the 200 ns time frame of the simulations, toward the edge of the large *trans*-compact global energy minimum, **I** samples *trans*-extended conformers in our 200 ns window of observations. In contrast, **III** samples a broad, diffuse distribution because the *cis*-compact geometry of the selected starting conformer lies near a local maximum of the H^P^ PMF; lacking stabilizing curvature at that position, it experiences no restoring force, and small thermal fluctuations are sufficient to drive the system away from the initial configuration. This behavior underscores that, in H^P^, the *trans*-compact basin is the most deeply stabilized, whereas the initial *cis*-compact structure used here does not relax into the neighboring *cis*-compact minimum within the time scale of the cMD simulations.

In the A^P^ system, conformers **I–II–III** exhibit broad sampling across the two-dimensional conformational space (Figure 4B, upper right), reflecting the inherently flexible nature of *apo* CaM under physiological ionic strength. The regions sampled by the unison of these simulations coincide with those observed in the MetaD PMF for A^P^ (Figure 2). These conformers diffuse extensively across torsion space, indicating that the A^P^ landscape contains shallow energetic curvature and permits facile reorientation of the lobes around a relatively pliable linker. Conformer **IV** also visits a compact minimum in A^P^, producing a narrow sampling cluster near (φ ≈ 150°, *l*_linker_ ≈ 15–20 Å). This behavior reflects strong intraprotein electrostatic clamps, which persist even in the absence of Ca^2+^.

In the H^L^ system, the cMD trajectories (Figure 4B, lower left) reflect the shallow, gently partitioned topology of the PMF, in which one region is a broad *cis* basin, accompanied by a secondary *trans*-compact minimum (Figure 3A). **IV** remains confined to this latter *trans*-compact well. By contrast, the other three conformers undergo basin-to-basin relaxation: **I**, initialized in the *trans*-extended quadrant, migrates toward the *cis* region with a somewhat shortened *l*_linker_. **II** spreads widely in both torsion angle and linker distance, sampling portions of the broad basin. **III**, although initialized in the *cis*-compact region, rapidly drifts toward the *trans*-compact basin. Because the *cis* region in H^L^ does not form a single sharply defined well but instead consists of three adjacent shallow minima separated by barely perceptible ridges, conformers readily slide toward neighboring basins, including the slightly higher-energy *trans*-compact state. These transitions, observed within the limited 200 ns time scale of the simulations, demonstrate that in low salt, the *holo* landscape is dominated by shallow internal structuring, allowing conformers to flow into alternative minima with minimal energetic resistance.

In the A^L^ system (Figure 4B, lower right), all starting conformers except **IV** disperse widely across the two-dimensional conformational space, consistent with the extremely shallow, weakly structured topology of its PMF (Figure 3B). Conformers **I**, **II**, and **III** rapidly lose memory of their initial states and diffuse throughout the extended region of the landscape, sampling a broad continuum of torsion angles and linker distances without settling into a distinct basin. This behavior reflects the absence of Ca^2+^-mediated intradomain stabilization, combined with reduced ionic screening, which together render *apo* CaM highly flexible and prone to large-amplitude domain reorientation. Conformer **IV**, initiated in the *trans*-compact corner of the landscape, remains near its starting position, consistent with the shallow *trans*-compact minimum on the A^L^ PMF. Overall, the A^L^ simulations demonstrate that low ionic strength amplifies the inherent structural pliability of *apo* CaM, yielding a nearly flat landscape dominated by a broad extended basin.

Overall, we find that conformers **I** and **IV** merit further scrutiny because they exhibit the strongest deviations from MetaD-predicted behavior and reveal condition-sensitive kinetic trapping. Conformer **I** unexpectedly becomes immobilized in H^L^, while **IV** remains rigidly compact across all environments. These anomalous dynamical signatures indicate that specific electrostatic and salt-bridge interactions may be reshaping their local landscapes, prompting more focused structural and energetic analyses.

### How Electrostatics Modulate the Stability of the Canonical CaM Architecture

Conformer **I** corresponds to the 3CLN structure which is the canonical dumbbell-shaped conformation that has served for decades as the defining structural archetype of CaM.^9,55^ This geometry underpins innumerable mechanistic models of CaM function, from descriptions of calcium sensing to target recognition, allosteric communication, and linker flexibility. Because 3CLN is so widely regarded as the “reference” state of CaM, it is crucial to establish whether this conformation is genuinely stable across physiologically relevant ionic and loading conditions, or whether it is a crystallographically selected state that becomes destabilized in solution. In our cMD simulations, **I** exhibits condition-dependent drift and even kinetic trapping in low salt, behaviors at odds with its canonical status (Figure 4). These observations motivate a more detailed mechanistic examination of the electrostatic environment and ion–protein interactions that modulate the stability of this historically central CaM conformation.

We first probe the electrostatic environment surrounding conformer **I** by calculating the electrostatic potential along its surface using the Adaptive Poisson–Boltzmann Solver (APBS) tool,^56^ enabling visualization of the local electrostatic landscape. Surfaces are calculated with standard biomolecular and solvent dielectric constants (2 and 78.5, respectively), probe radius of 1.4 Å and with ionic compositions matched to each simulation condition of Table 1. Consistent with CaM’s strongly negative net charge at physiological pH (−23 for *apo*, −15 for *holo*), the protein surface is dominated by negative potential as shown in Figure 5A. However, the extent and spatial distribution of these negative regions depend strongly on ionic strength. We find that physiological salt partially neutralizes the electrostatic landscape, producing neutral patches in *apo* CaM and even small positive regions in the *holo* form mostly around regions of Ca^2+^ coordination. Low-salt conditions, in contrast, yield a uniformly negative surface that is expected to attract and retain counterions (K^+^) more strongly.

**Figure 5:**
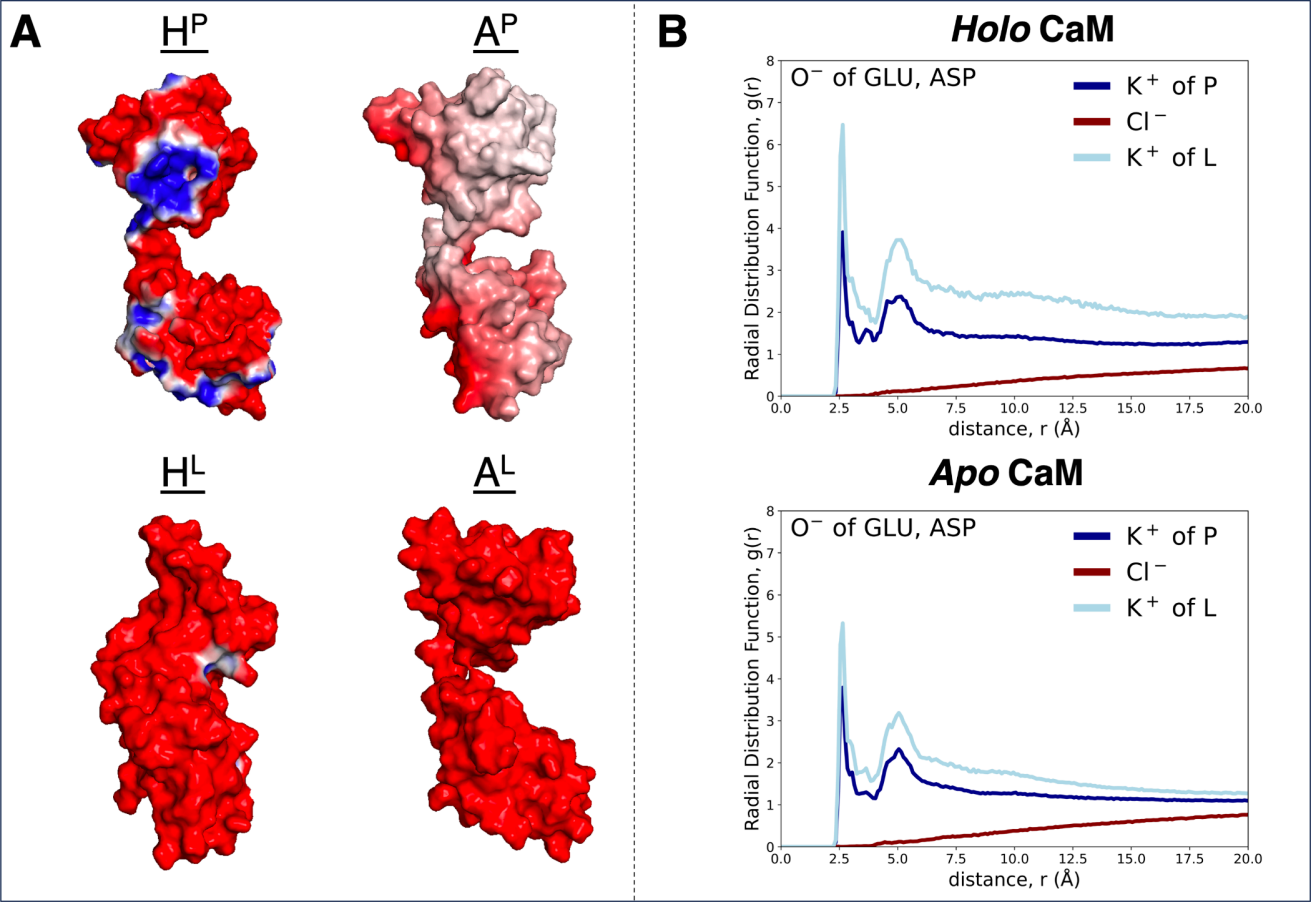
(A) Electrostatic isocontours of conformer **I** drawn at ± 1 *k*_B_ *T/e*. Blue, red, and white regions represent positive, negative, and neutral electrostatic potentials, respectively, along the surface of the protein. (B) Radial distribution function, *g*(*r*), calculated between the C_β_ atoms of negatively charged residues and the surrounding K^+^ and Cl^–^ ions for the 200 ns-long simulations started from conformer I.

To quantify this ion association directly, we computed radial distribution functions (RDFs) between negatively charged side chains and surrounding ions for all simulation conditions (Figure 5B; for broader comparison see Figure S5). Across all conformers, a first coordination shell of K^+^ ions at 2–4 Å and a second one at 4–6 Å are consistently observed. Strikingly, more K^+^ ions approach the CaM surface under low-salt conditions, even though the number of K^+^ ions present in the physiological systems is significantly larger. This effect is robust and observed regardless of Ca^2+^ loading. The explanation becomes clear when examining Cl^–^ ion distributions, which under physiological salt form a diffuse co-ion layer that competes with K^+^ for proximity to the protein surface, weakening the electrostatic focusing of counterions. In low salt, however, because only K^+^ ions were added to neutralize the system in these simulations (Table 1), the absence of co-ions leads to counterion condensation, producing tighter K^+^ clustering around acidic residues.

These ion-distribution trends are consistent with the electrostatic potential maps of Figure 5A such that physiological salt reduces and redistributes surface charge, while low salt enhances large contiguous negative patches that recruit more K^+^ ions. Such counterion accumulation can modulate conformational dynamics in two opposing ways: **(i)** by stabilizing compact electrostatically frustrated arrangements through local charge compensation, or **(ii)** by acting as “electrostatic lubricants” that weaken salt bridges and interdomain contacts, thereby enabling conformational rearrangements.

For **I**, the data suggest that both effects operate depending on ionic strength. Under physiological salt, weaker ion clustering and partial surface neutralization promote broader conformational mobility, consistent with the more diffuse sampling in H^P^ and especially in A^P^ (Figure 4). In the latter, the absence of Ca^2+^-mediated intradomain stabilization allows the two lobes to reorient more freely around the linker, yielding the widest coverage observed starting from the canonical dumbbell geometry. In fact, this condition even opens a route for this conformer to escape to the compact states exemplified by the 1PRW crystal structure. The conformational flip between 3CLN and 1PRW has been scrutinized in our earlier work where turning off the charge in E31 located in one of the EF-hand motifs enabled the compaction but not the rotation,^30^ Under low salt, however, dense K^+^ association reinforces local negative patches and enhances electrostatic steering, which, when combined with rigid Ca^2+^-loaded EF-hands in H^L^, promotes kinetic trapping of **I** in the shallow pockets of its landscape. In *apo* low-salt conditions (A^L^), the absence of EF-hand stabilization reduces this effect, and the conformer samples a somewhat broader region compared to H^L^.

These analyses demonstrate that the stability of the canonical 3CLN conformation is not intrinsic but emergent, arising from a subtle balance among Ca^2+^-dependent intradomain rigidity, the redistribution of surface potential by ionic screening, and the local clustering of counterions at low ionic strength.

### How Salt-Bridge Networks Shape the Stability of the Compact CaM Architecture

Although counterion condensation introduces two competing effects, **(i)** stabilization of compact charge-frustrated regions and **(ii)** electrostatic lubrication that weakens salt bridges, their relative influence differs across conformers. Thus, while the same electrostatic processes operate across all conformers (Figure S5), the balance between stabilizing versus lubricating depends on the underlying geometry and packing of each state, which is reflected in the shifts in their RDF profiles. In the canonical dumbbell (3CLN), lubrication dominates, producing broad dispersion under physiological salt and kinetic trapping under H^L^. Similarly, the *cis*-extended **II** also lacks a compact interdomain interface minimizing the stabilizing effects, and its behavior across conditions is similar to conformer **I**. In contrast, the *cis*-compact **III** sits on a marginal ridge of all PMFs, where counterions preferentially weaken rather than reinforce interdomain contacts, facilitating its migration into neighboring basins across all conditions.

In contrast, the *trans*-compact CaM architecture, represented in our simulations by **IV** that remains compact across environments, possesses a preorganized interdomain salt-bridge network that creates a stabilizing electrostatic clamp. This geometry uniquely benefits from the stabilizing effect of counterion condensation, as displayed by the largest K^+^ recruitment on the surface in both *apo* and *holo* forms, particularly in the low ionic strength conditions (Figure S5). To dissect the molecular basis of this rigidity, we quantified salt-bridge occupancies across conditions (Table S1) and traced the time evolution of interdomain charge–charge interactions for persistent salt bridges specific to this compact state (Figure 6A).

**Figure 6:**
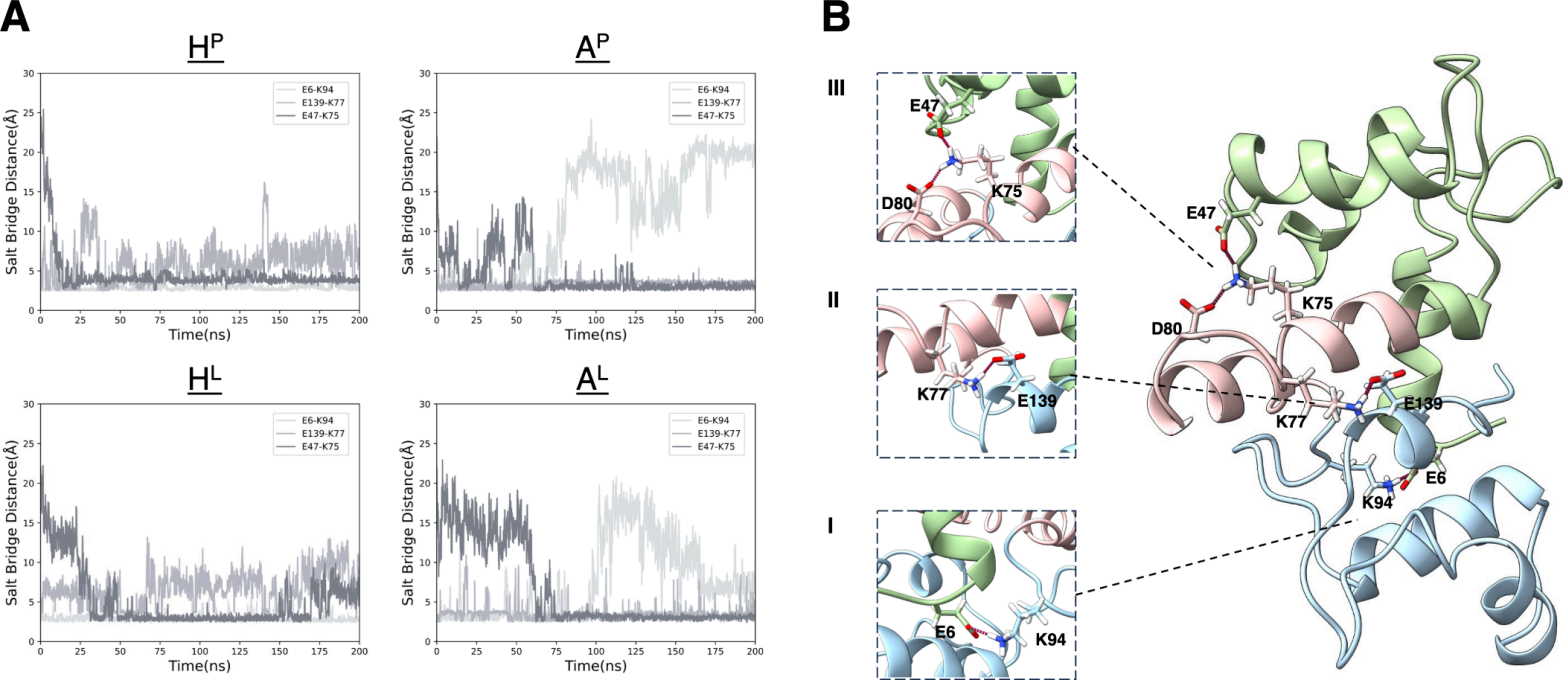
(A) Changes in unique salt bridge interactions of **IV** throughout the cMD trajectories for all systems, and (B) structural representations show salt bridge interactions identified in **IV** (snapshot selected from A^P^ simulation).

Three key electrostatic contacts underpin the stability of this compact architecture. First, the long-range salt bridge between E6 (N-lobe) and K94 (C-lobe) draws the two lobes inward and bends the helical linker (Figure 6B-**I**). This bridge is strong and persistent throughout the 200 ns simulations in both *holo* systems (H^P^, H^L^), the distance fluctuating between 3.2–5 Å (Figure 5A). It weakens earlier in the A^P^ trajectory and becomes intermittently labile in A^L^, reflecting the absence of Ca^2+^-stabilized EF-hand geometry. Second, the K77–E139 interaction couples the central linker to the C-lobe (Figure 6B-**II**). Though this bridge alternates between formed and broken states in H^P^ and H^L^, it remains remarkably stable in *apo* environments (A^P^ and A^L^), consistently residing near 3.2 Å. Third, the E47–K75 salt bridge (Figure 6B-**III**) forms only after the first two interactions have been established. In *apo* systems, it stabilizes after ∼60 ns. In *holo* systems, it is established earlier on (<30 ns in these cMD simulations) and in H^L^ it becomes intermittently unstable after 170 ns, cycling between bound and unbound states.

These three bridges cooperatively contract the linker region and draw the lobes together. The cumulative effect is a multivalent interdomain clamp that restricts rotational freedom and enforces a compact geometry even under conditions where the free-energy landscape does not strongly favor it. The persistence of this compact state across ionic strengths and Ca^2+^-loading regimes therefore arises not from global landscape features but from the robustness of these salt-bridge networks, which act as internal stabilizers against both electrostatic lubrication and energetic flattening.

Together, these results show that compact CaM architectures benefit uniquely from counterion-modulated stabilization. Whereas extended or marginally compact states respond to ionic screening primarily through enhanced flexibility and basin-to-basin drift, the compact architecture is reinforced by a pre-existing interdomain electrostatic scaffold that remains partially intact across salt concentrations and Ca^2+^-loading regimes. In the A^P^ system in particular, ionic “lubrication” is insufficient to release the compact state: the same screening that softens long-range interactions leaves the short-range, multivalent E6–K94/K77–E139/E47–K75 clamp largely intact, so that concerted disruption of all contacts remains too rare to permit escape from this kinetically trapped configuration on the 200 ns time scale of the simulations. Although screening weakens individual interactions, it seldom breaks the entire network simultaneously. Since large-scale interdomain rearrangements require barrier-crossing events that cMD is unlikely to sample on these time scales, the compact state persists as the most kinetically rigid species in our simulations, even in conditions such as A^P^ and A^L^ where MetaD reveals no corresponding thermodynamic minimum, reflecting kinetic trapping enforced by the robust interdomain electrostatic network.

### How Cellular Context Selects CaM Conformations

CaM acts as a central interpreter of calcium signals across compartments that differ dramatically in ionic composition, calcium dynamics, and membrane architecture. Although it is primarily an intracellular protein found in the cytosol and nucleus,^13^ CaM also interacts with proteins in various calcium-regulating compartments^57^ (Figure 7A) where it not only regulates calcium levels but also plays a crucial role in calcium signaling by undergoing conformational changes that enable interactions with these proteins within organelles.^13^ This ability to regulate both calcium homeostasis and signaling demonstrates how CaM dynamically adjusts its structure to accommodate different binding partners. Its diverse localization, combined with its structural adaptability, allows it to function as a versatile regulator of calcium-dependent processes. This context-dependence makes CaM an ideal system to connect the MetaD-derived conformational landscapes to real biological function.

**Figure 7:**
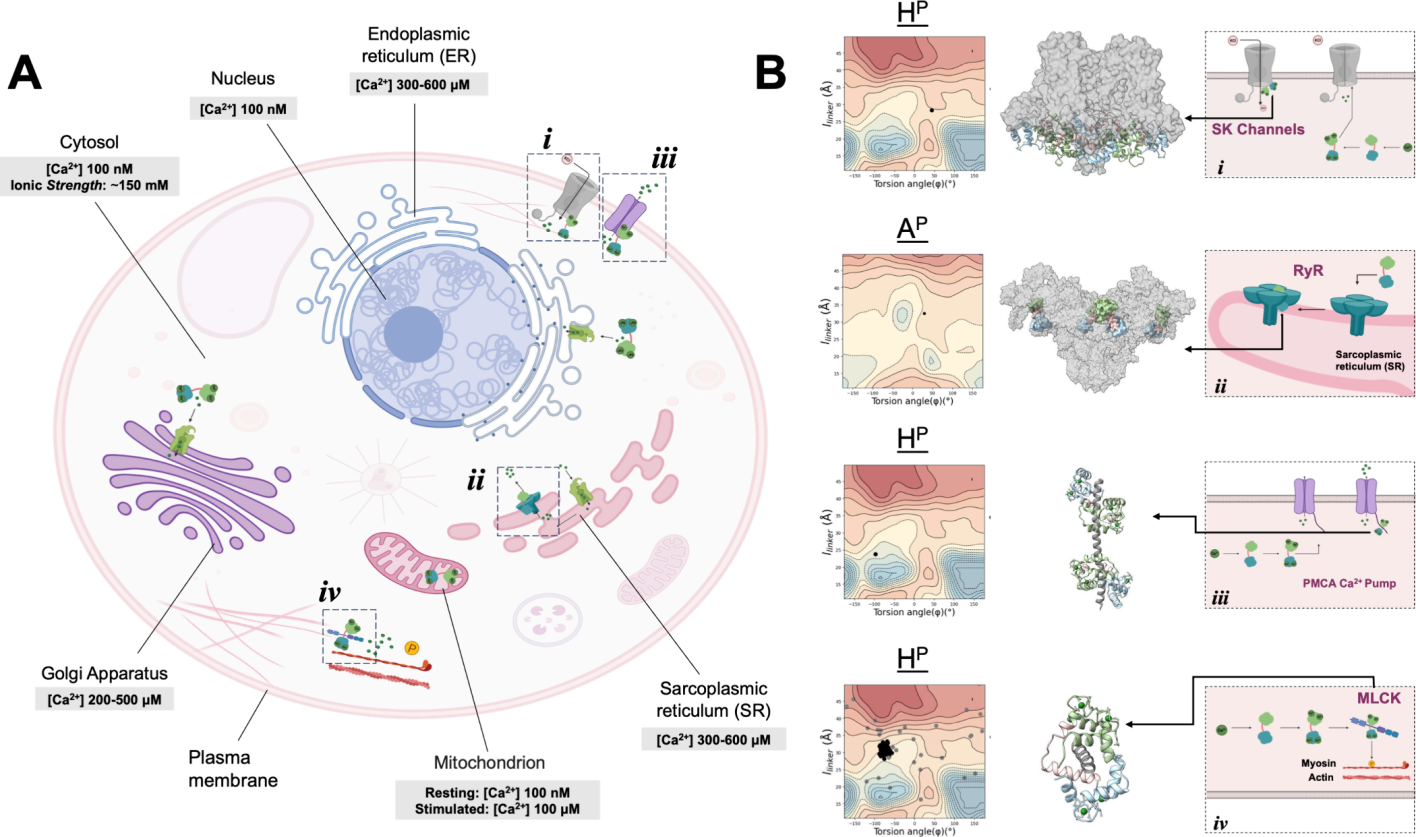
(A) A diagram of a eukaryotic cell shows the main organelles, typical calcium concentration distribution across compartments (created with biorender.com), and proteins located in different parts of the cell whose functions are regulated by CaM; (B) comparative conformational landscapes of CaM from MetaD simulations, overlaid with conformations of CaM from experimental structures with binding partners. Additionally, schematic illustrations depict how CaM interacts with these targets. CaM binding is shown for SK channel located in the plasma membrane (PDB ID: 6CNN) **(** *i* **)**, the RyR at the SR (PDB ID: 6JI8) **(** *ii* **)**, PMCA located in the plasma membrane (PDB ID: 4AQR) **(** *iii* **)** and MLCK in the cytoplasm (PDB IDs: 2K0F shown in black and 1MUX shown in gray) **(** *iv* **)**.

To probe the functional significance of the conformational states sampled in our simulations, we projected experimental CaM–protein complex structures from multiple organelles onto our (*l*_linker_, φ) space (Figure 7B). We find that a coherent picture emerges whereby target-bound CaM overwhelmingly adopts *trans*-extended geometries, even when the target free, Ca^2+^-loaded protein favors a *cis*-compact state thermodynamically. This demonstrates that target engagement reshapes the CaM free-energy landscape, selecting conformations that are not the lowest-energy states in solution but become functionally stabilized through protein–protein interactions.

We first exemplify the conformational behavior of CaM in the plasma membrane. Here, CaM regulates both ion channels and Ca^2+^ extrusion machinery. Small-conductance calcium-activated potassium (SK) channels are membrane proteins that facilitate K^+^ flow across the cell membrane^58^ (Figure 7A, ***i***). They are activated by intracellular Ca^2+^ but remain voltage-independent. Each SK channel tetramer associates constitutively with four CaM molecules,^59^ which act as built-in Ca^2+^ sensors. Upon Ca^2+^ binding, *holo* CaM fits into the SK channel cavity (Figure 7B, ***i***). This interaction induces conformational changes that open the pore, allowing K^+^ ions to pass through.^58,59^ Without CaM, SK channels become inactive as they cannot respond to intracellular Ca^2+^ concentration changes. Cryo-EM structure shows that *holo* CaM adopts a *cis*-extended geometry when bound to SK channels, precisely the region sampled in the shallow halo surrounding the H^P^ basin in our MetaD surface (Figure 7B, ***i***). This conformation places both lobes in a configuration that can transmit Ca^2+^-dependent structural changes directly to the channel gating ring, providing a mechanistic explanation for how Ca^2+^ triggers pore opening.

On the sarcoplasmic reticulum (SR) ryanodine receptors (RyRs) are intracellular channels that mediate Ca^2+^ release into the cytoplasm^60^ (Figure 7A, ***ii***). RyRs integrate both Ca^2+^-dependent and Ca^2+^-independent modes of CaM regulation.^61^ *apo* CaM suppresses RyR activity to prevent excessive Ca^2+^ leakage, while calcium-bound CaM fine-tunes RyR function during excitation-contraction coupling for controlled release.^62^ Cryo-EM structure of RyR bound to *apo*-CaM reveals that the N-lobe of CaM is positioned in the upper part of a cleft in RyR’s helical domain, while the C-lobe rests at the bottom, near the handle and central domains (Figure 7B, ***ii***). When projected onto our MetaD landscapes, this structure aligns with the *cis*-extended minimum of the A^P^ surface, indicating that *apo* CaM binds RyR in a conformation that is naturally stable under physiological salt. This reinforces the established model in which *apo* CaM suppresses RyR leak by maintaining a tensioned, extended geometry stabilizing a closed channel.

In the plasma membrane, CaM also interacts with Ca^2+^-ATPase (PMCA), a transport protein that moves Ca^2+^ from the cytosol to the extracellular space, maintaining low intracellular Ca^2+^ levels^63,64^ (Figure 7A, ***iii***). When Ca^2+^ levels rise, *holo* CaM undergoes a conformational change and wraps around the C-terminal binding domain of PMCA, activating the pump and increasing Ca^2+^ efflux from the cell.^65^ Similar to SK, in its complex with PMCA, *holo* CaM adopts an arrangement that wraps around an 18–1 regulatory peptide (Figure 7B, ***iii***). This binding-induced compaction occurs in the second compact basin on the (*l*_linker_, φ) landscape of *holo* CaM, suggesting that PMCA binding stabilizes a conformation sampled on the PMF.

In the cytoplasm, one well-known interaction partner of CaM is myosin light chain kinase (MLCK), a cytoplasmic enzyme associated with stress fibers and the cleavage furrow during cell division^66,67^ (Figure 7A, ***iv***). It phosphorylates the regulatory light chain of myosin II, activating myosin for muscle contraction and other actomyosin-dependent processes.^66^ MLCK activity is significantly enhanced by CaM binding, whereas in its absence, the kinase remains largely inactive, impacting cellular signaling.^66^ Ca^2+^-loaded CaM activates MLCK by wrapping around a helical target peptide. In the NMR ensemble of the complex (PDB ID: 2K0F), CaM lies near the *cis*-extended region (Figure 7B, ***iv***), with its C-lobe engaging the peptide and the N-lobe clamping around the target.^10^ This architecture lies well outside the deep *trans*-compact well of the H^P^ surface. Thus, peptide binding must reshape the CaM energy landscape, overriding the intrinsic *trans*-compact preference of calcium-loaded CaM and stabilizing an elongated geometry necessary for MLCK activation. The agreement of experimental structures with the accessible but nondominant MetaD regions supports a model in which CaM’s intrinsic landscapes set the repertoire of possible regulatory states, while binding partners select and stabilize specific ones for function.

In contrast, overlaying the 30 conformers in the 1MUX NMR ensemble, having a structural and functional mimic of the CaM-binding region of MLCK,^68^ onto the MetaD-derived landscape (Figure 7B, ***iv***) shows that the *holo* CaM structures in this complex populate even further extended conformations with *l*_linker_ > 35 Å. Rather than occupying the deep *trans*-compact minimum that characterizes free *holo* CaM, the ensemble represents extended states that are only weakly sampled in our simulations. By occupying MLCK-recognition pockets, W-7 forcibly prevents lobe–lobe collapse and stabilizes extended CaM geometries, effectively overriding the intrinsic *trans*-compact preference of Ca^2+^-loaded CaM. Thus, the 1MUX ensemble provides a clear structural demonstration that MLCK-like target engagement can recruit *holo* CaM into elongated states located outside its intrinsic energetic minimum, consistent with the extended regions partially accessed in our MetaD surface.

The contrast between the 1MUX and 2K0F ensembles highlights how different target peptides sculpt distinct regions of the CaM energy landscape. In 1MUX, the flexible W-7 peptide provides only weak geometric constraints and allows *holo* CaM to populate a wide continuum of elongated states, spanning both *cis*- and *trans*-extended geometries. In 2K0F, however, the CaM-binding partner is a preorganized amphipathic helix with a fixed hydrophobic register, enforcing a single interdomain orientation and collapsing the ensemble onto a narrow band in the *cis*- region. Thus, the breadth or specificity of CaM’s extended conformational states is determined not only by its intrinsic landscape but by the structural rigidity and anchoring pattern of the bound target peptide.

These cross-compartment examples highlight unifying trends for CaM conformational selection: First, *apo* CaM functions predominantly in extended geometries, consistent with our A^P^ and A^L^ free-energy surfaces. This underlies its regulatory roles in preventing aberrant Ca^2+^ release (RyR). Second, Ca^2+^ loading introduces a deep compact basin, which is biologically relevant for peptide-free CaM but is rarely used directly for target binding. Instead, Ca^2+^-loaded CaM typically transitions into less compact regions upon engaging partners like SK channels, PMCA, and MLCK. This engagement may also help release the deeply stabilizing linker – lobe salt bridges of CaM (Figure 6). Third, target binding consistently recruits CaM into higher-lying regions of the MetaD landscape, demonstrating that functional conformations are often not the global thermodynamic minima of the isolated protein. Instead, protein–protein contacts stabilize geometries that are subdominant or transient in solution.

In sum, ligand binding reshapes CaM’s charge distribution and steric constraints in ways that disrupt compact-state salt-bridge stabilization and redirect the protein into specific elongated conformations drawn from its intrinsic solution-phase landscape. Interpreting simulations with experimental structures illustrates clearly how CaM’s environment, i.e., ionic strength, calcium loading, organellar context, and binding partner, collectively reshapes its conformational ensemble. The result is a dynamic, context-sensitive regulator capable of executing distinct tasks across the cell.

## Conclusions and Future Perspectives

CaM’s versatility as a calcium sensor emerges from a finely tuned interplay between Ca^2+^ binding, ionic strength, and intrinsic electrostatics, all of which reshape a structured yet adaptable conformational landscape. By combining well-tempered MetaD with classical MD simulations, and interpreting our findings with experimentally determined structures, we show that CaM’s conformational ensemble is not merely flexible but systematically regulated by environmental conditions and requirements for engagement with specific targets.

Under physiological ionic strength (Figure 2), CaM is driven into deeply stabilized basins. Reducing the ionic strength (Figure 3) greatly flattens the free-energy surfaces for both states, lowering barriers and enabling facile interconversion. Yet, this flattening simultaneously promotes spurious electrostatic trapping, as strong K^+^ condensation around acidic residues stabilizes local minima that are bypassed in MetaD but kinetically persistent in unbiased simulations (Figure 4). Comparing MetaD and cMD results reveals that while MetaD exposes CaM’s thermodynamic possibilities, cMD reveals which of those states are kinetically stable. Many higher-lying MetaD basins dissipate under unbiased dynamics, while one structure, the *trans*-compact conformer, remains strikingly immobile across all conditions due to a network of salt-bridges (Figure 6). This rigid architecture underscores how short-range electrostatics can override broader energetic trends.

A coherent picture emerges when the conformational landscapes of free CaM are considered alongside ligand-bound structures across cellular compartments. In fact, overlaying experimentally observed CaM–target complexes onto our landscapes highlights that functional CaM conformations often lie outside thermodynamic minima (Figure 7). In solution, ionic strength modulates the ease with which CaM moves between states by reshaping counterion condensation and the stability of interdomain contacts (Figure 5). Target engagement shifts this balance not by altering ionic composition but by reorganizing CaM’s charge distribution and steric geometry. Binding partners, whether flexible antagonists for MLCK such as W-7 in 1MUX or highly structured regulatory helices as in 2K0F, or membrane-associated peptides from SK channels and PMCA, bury acidic pockets, generate new hydrophobic anchors, and sterically block formation of the compact-state salt-bridge clamp. As a result, the multivalent electrostatic scaffold that stabilizes the *trans*-compact minimum is dismantled, and CaM is redirected into elongated conformations that match the functional geometry required by each target. Thus, ligand binding selectively stabilizes higher-lying but readily accessible regions of CaM’s intrinsic energy landscape, allowing the protein to adopt distinct regulatory states across different cellular contexts.

Together, these results provide an integrated view of CaM as a protein whose conformational preferences are not fixed but emergent outcomes of electrostatics, ion composition, and target-induced remodeling. They also clarify long-standing discrepancies between crystallographically determined states, NMR ensembles, and solution-phase biophysical measurements, showing how each corresponds to a different region of the underlying multidimensional landscape.

We note that while the rotation–linker distance CV pair offers clear interpretability and successfully recapitulates known conformers, it remains a reduced projection of CaM’s high-dimensional dynamics. Exploring alternative collective variables in future studies, including machine-learning-derived CVs, may reveal additional slow modes or refine the boundaries between basins, although such approaches may reduce the direct physical interpretability that is central to the present analysis. Future work could also focus on identifying transition pathways between conformers, incorporating experimental restraints from FRET or MS, or simulating explicit target-binding events to map how partner engagement deforms the energy landscape. Ultimately, the new conformers and mechanistic insights identified here provide a foundation for predictive modeling of CaM regulation and for designing synthetic peptides or mutations that tune CaM’s conformational equilibria.

## Supplementary Material



## Data Availability

Original code for producing the results of this study is deposited on GitHub at the following repository: https://github.com/midstlab/Tayhan_2026. All classical MD (cMD) trajectories as well as complete outputs of cMD and metadynamics (MetaD) simulations generated in this work are available on Zenodo (https://zenodo.org/records/18293098).

## Notes

Figure S1: RMSD profiles of initial cMD simulations; Figure S2: Torsion angle (φ) – linker end-to-end distance (*l*_linker_) plots; Figure S3: MetaD trajectories for the two selected CVs; Figure S4: Energy landscapes sampled by MetaD simulations; Figure S5: Radial distribution function, *g*(*r*), between C_β_ atoms of the negatively charged residues and KCl ions for all conformers; Table S1: Salt Bridge occupancies exceeding 20% (PDF)
