# Supplementary material for: Functional Calmodulin States Are Selected from an Electrostatically Tuned Free Energy Landscape
Source: J Chem Inf Model. 2026 May 16;66(11):6602–16. doi: 10.1021/acs.jcim.6c00532 (PMC13250904; doi:10.1021/acs.jcim.6c00532)
Supplement: Supplementary file 1 [file ci6c00532_si_001.pdf]

Supplementary Material for

Functional Calmodulin States are Selected from an Electrostatically Tuned Free Energy Landscape

Busra Tayhan [a], Sila Horozoglu [a], Ali Rana Atilgan [a], Canan Atilgan [a*]

[a] Faculty of Engineering and Natural Sciences, Sabanci University, Tuzla 34956 Istanbul, Türkiye

* E-mail: canan@sabanciuniv.edu

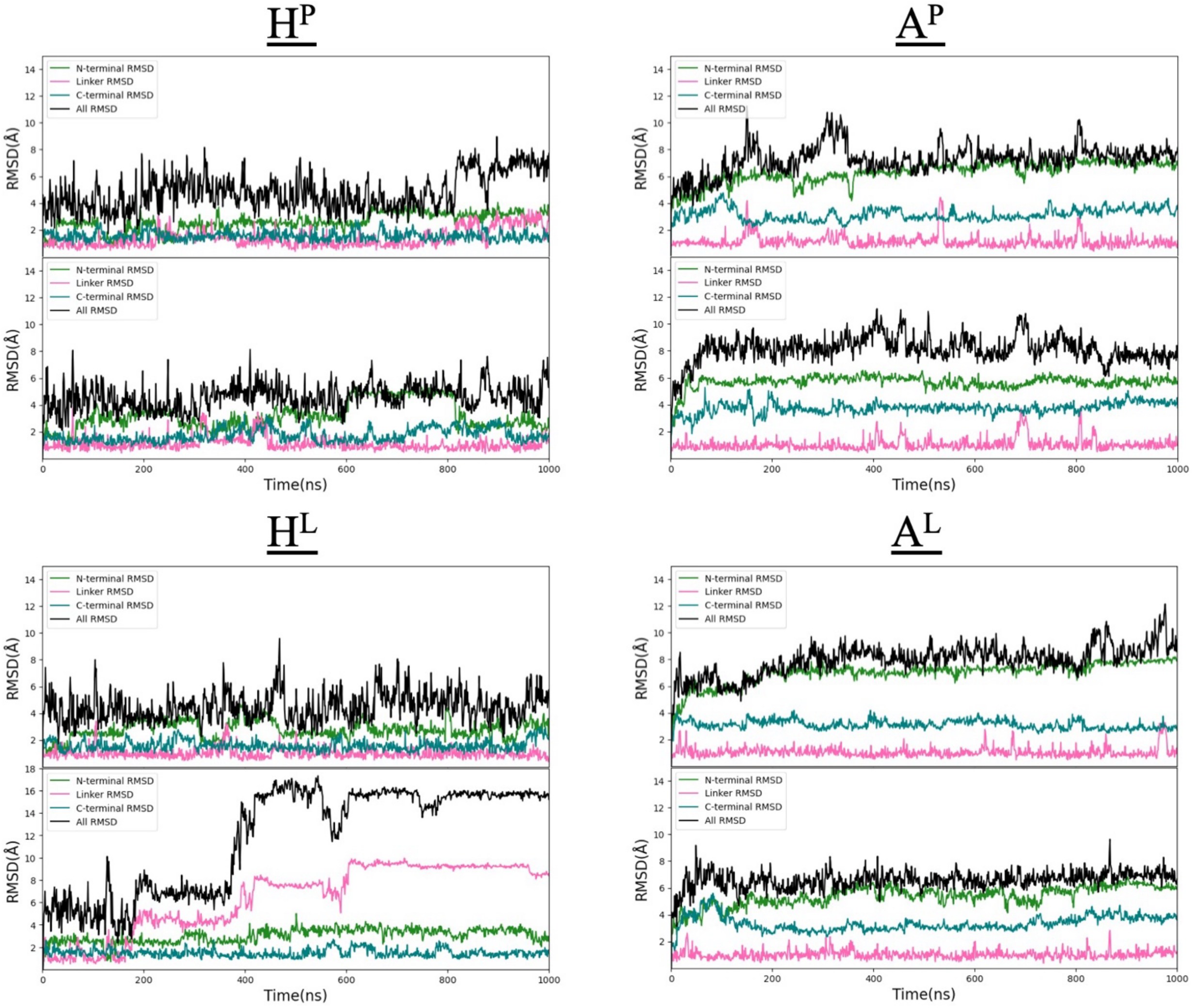

Figure S1. RMSD graphs of two independent 1 μs long simulations of CaM in different environmental conditions; H^P, A^P, H^L and A^L. N-terminal domain, C-terminal domain, flexible linker region, and overall RMSD are depicted in green, blue, pink, and black, respectively. The colors of the regions correspond to the coloring displayed in **Figure 1**.

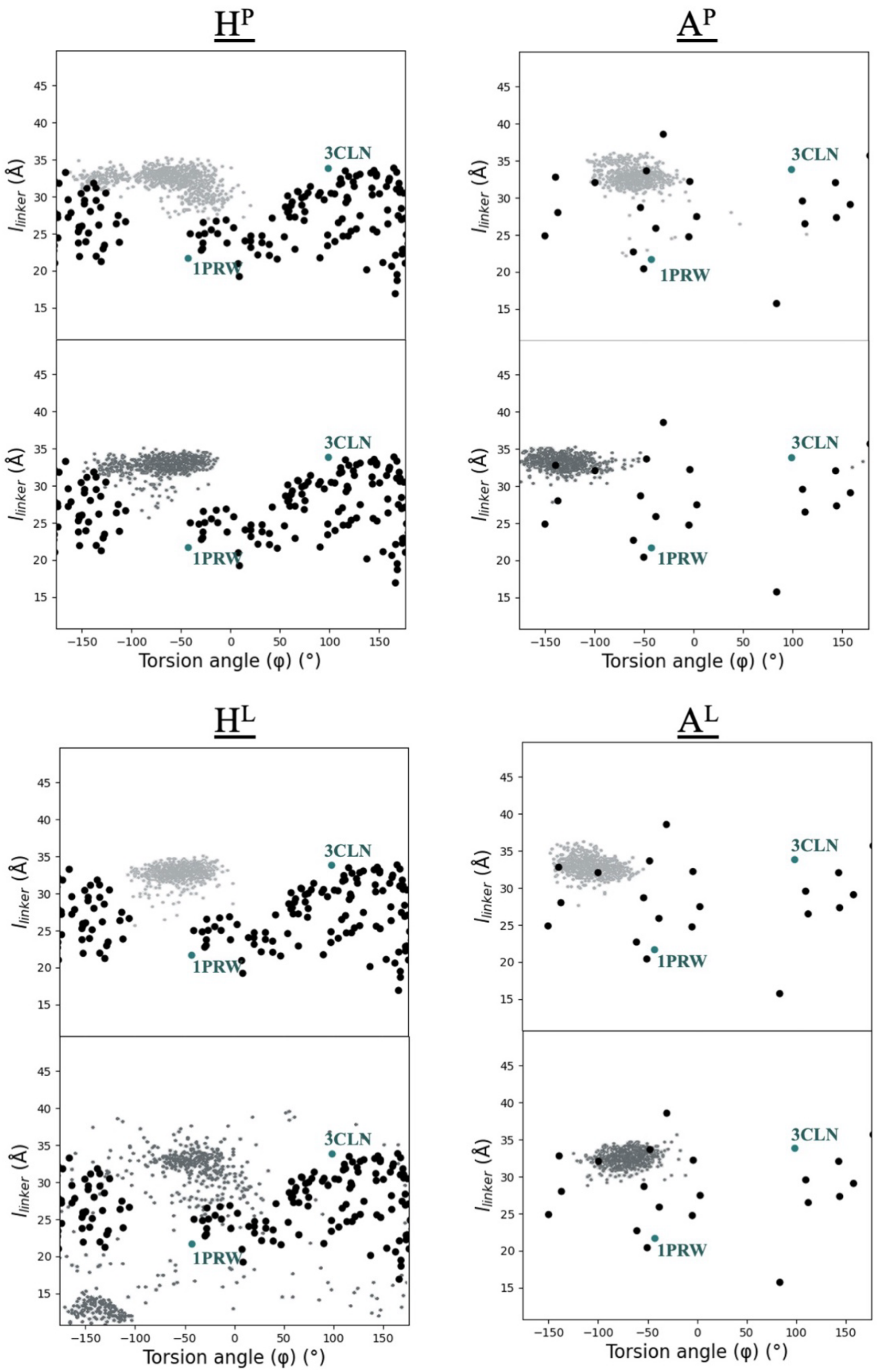

Figure S2. Torsion angle (*φ*) – linker end-to-end distance (l_{linker}) plots from duplicate cMD trajectories (light gray/dark gray small dots) under the four environmental conditions studied, all started from the 3CLN initial structure. For reference, experimental structures are also overlaid with the larger dots: Black ones represent NMR structures determined for *holo* (2K0E; 160 conformers) or *apo* CaM (6Y95; 20 conformers); in teal color are two labelled crystal structures.

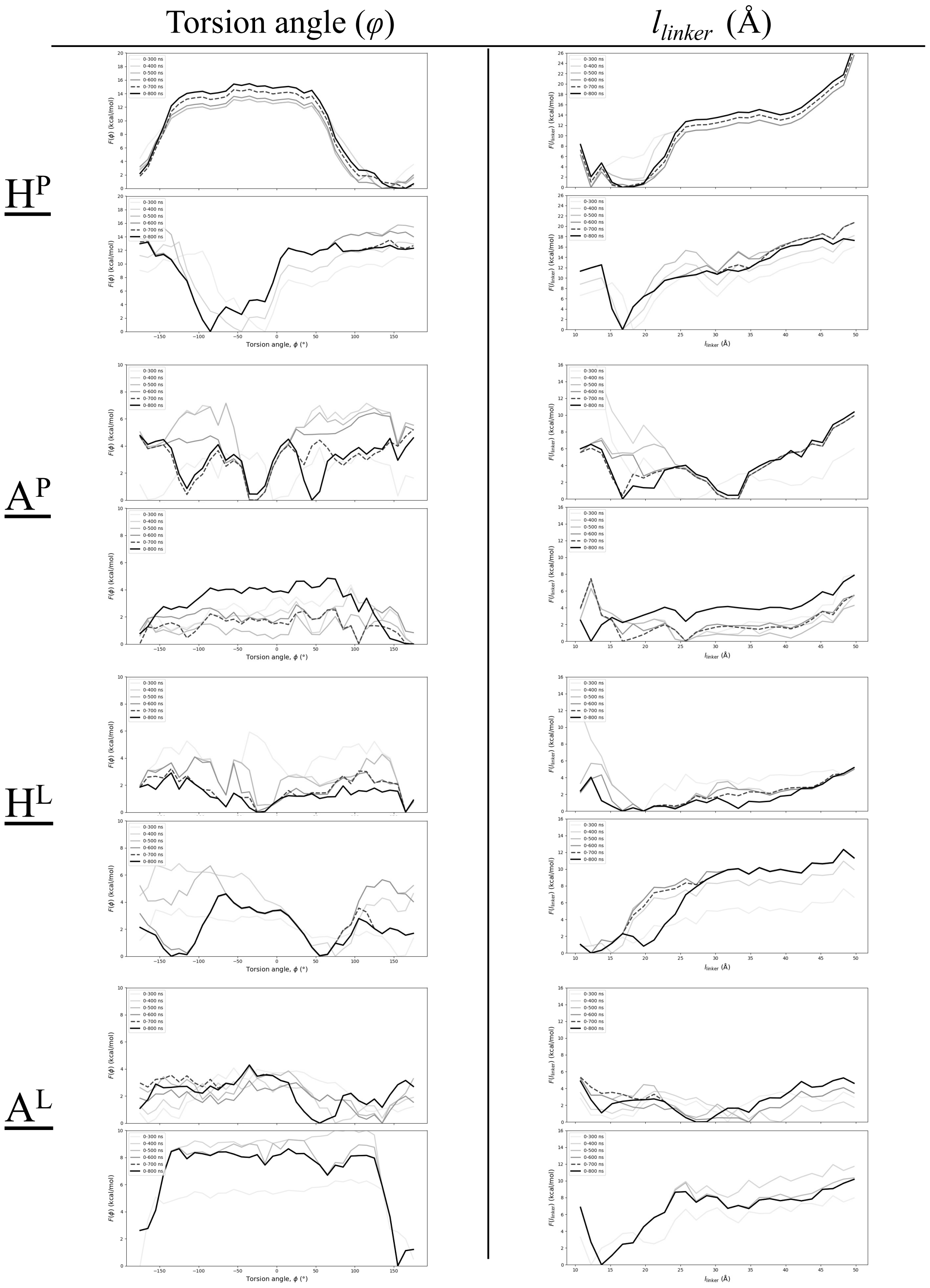

Figure S3. Cumulative one-dimensional free-energy profiles of duplicate simulations across all systems, projected onto the torsional angle (°), and linker end-to-end distance (Å) , at cumulative simulation times of 300, 400, 500, 600, 700, and 800 ns. Curves are shown from lighter to darker gray with increasing simulation time.

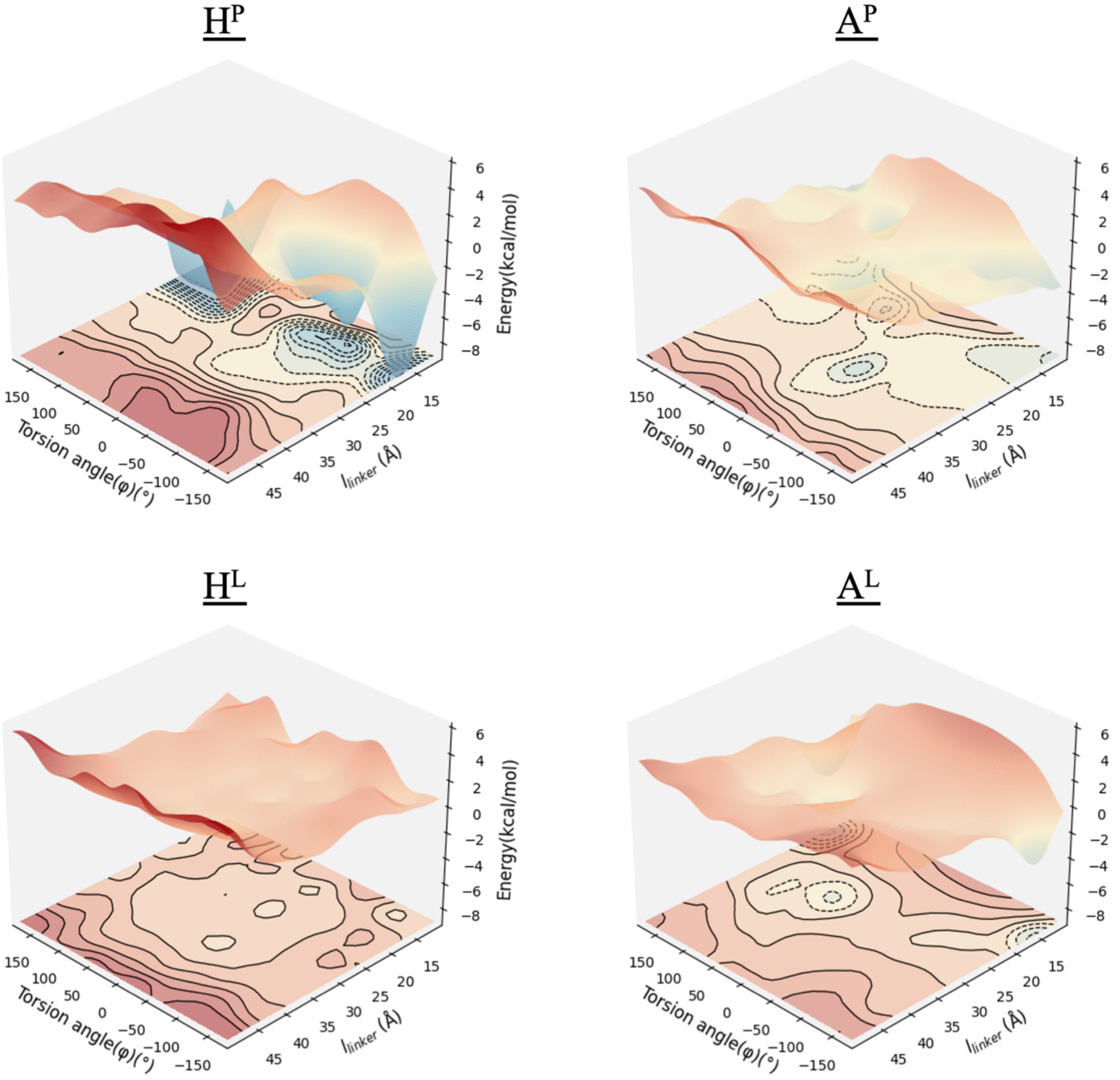

Figure S4. Energy landscapes sampled by MetaD simulations for *holo* CaM under **a** physiological and **b** low salt conditions, and for *apo* CaM under **c** physiological and **d** low salt conditions

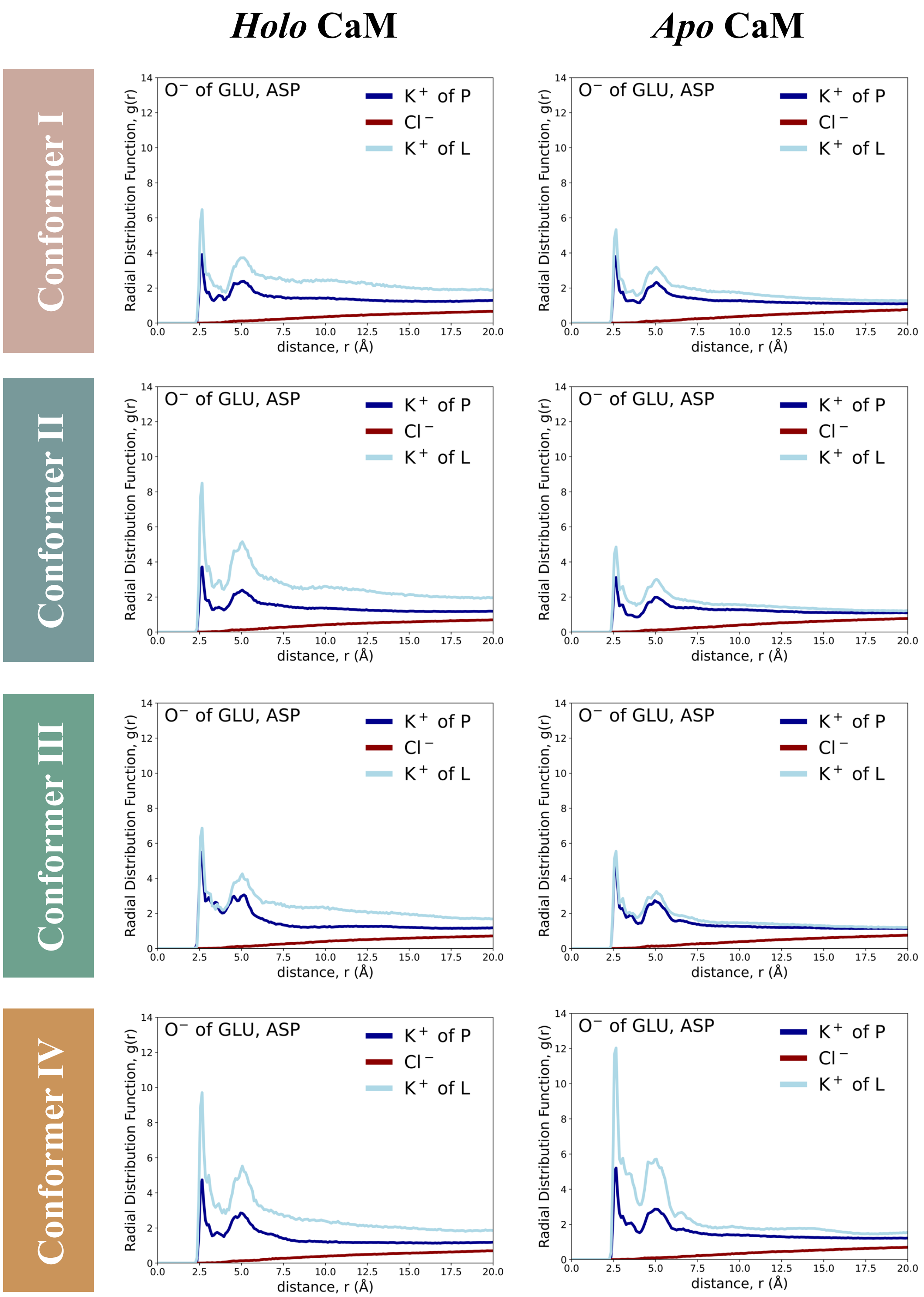

Figure S5. Radial distribution function, $g(r)$, between C_β atoms of the negatively charged residues and KCl ions for all conformers. Potassium ions under low salt conditions are shown in light blue (K^+ of A^L and H^L), while potassium ions under physiological salt are shown in dark blue (K^+ of A^P and H^P). Chloride ions under physiological salt conditions are shown in red (Cl^-).

Table S1. Salt Bridge occupancies exceeding 20% for all conformers*

Conformer I

H^P Interaction	H^P Occupancy (%)	A^P Interaction	A^P Occupancy (%)	H^L Interaction	H^L Occupancy (%)	A^L Interaction	A^L Occupancy (%)
E54-R74	54	E31-K21	35	E47-K77	58	E31-K21	24
E84-K115	36	E47-K77	32	E54-R74	44	E54-R74	72
E104-K94	31	E54-R74	50	E82-K77	50	E82-K77	28
E120-K75	23	E87-R90	28	E83-R86	29	E87-R90	24
D122-R106	37	E104-K94	40	E83-R90	25	E104-K94	28
		D50-K77	28	E87-R90	48	E139-R86	20
		D78-K75	25	E104-K94	46	D22-K21	20
		D95-K94	24	D95-K94	27	D122-R106	49
		D122-R106	46	D122-R106	61		
		D122-R126	28	D131-K30	39		
				D133-K30	30		

Conformer II

H^P Interaction	H^P Occupancy (%)	A^P Interaction	A^P Occupancy (%)	H^L Interaction	H^L Occupancy (%)	A^L Interaction	A^L Occupancy (%)
E31-K30	36	E47-K75	84	E31-K21	26	E31-K21	29
E45-K30	22	E54-R74	24	E45-K30	55	E47-K75	67
E54-R74	31	E82-K77	30	E54-K75	77	E54-R74	64
E82-R86	22	E82-R86	32	E83-R74	27	E104-K94	60
E84-K75	50	E83-R86	34	E87-R90	22	D22-K21	34
E84-K77	24	E104-K94	49	E123-R126	23	D78-K75	34
E114-K30	53	E139-R86	21	E139-K75	33	D122-R106	71
D122-R106	22	D122-R106	68	E140-R74	21		
				D80-R74	21		
				D80-K75	27		
				D122-R106	57		

Conformer III

H^P Interaction	H^P Occupancy (%)	A^P Interaction	A^P Occupancy (%)	H^L Interaction	H^L Occupancy (%)	A^L Interaction	A^L Occupancy (%)
E31-K21	26	E47-K77	26	E31-K21	45	E31-K21	36
E47-K77	47	E54-R74	26	E45-R37	29	E47-K77	27
E54-R74	31	E84-K75	45	E47-K77	45	E54-R74	40
E82-K77	20	E87-R90	54	E54-R74	29	E87-R90	23
E83-R86	21	E104-K94	40	E82-R86	20	E104-K94	36
E87-R90	33	E139-K77	37	E87-R90	29	D95-K94	47
D22-K21	28	D78-K75	43	E139-R74	22	D122-R106	72
D122-R106	78	D80-K75	28	D50-K77	50		
		D95-K94	45	D78-K77	48		
		D122-R106	57	D122-R106	32		
		D122-R126	21				

Conformer IV

H^P Interaction	H^P Occupancy (%)	A^P Interaction	A^P Occupancy (%)	H^L Interaction	H^L Occupancy (%)	A^L Interaction	A^L Occupancy (%)
**E6-K94**	**88**	**E6-K94**	**25**	**E6-K94**	**83**	**E6-K94**	**36**
E7-K94	41	**E47-K75**	**66**	E31-K21	23	E7-K94	23
E14-K21	21	E54-R74	71	**E47-K75**	**62**	E31-K21	48
E54-R74	64	E87-R90	49	E54-R74	62	**E47-K75**	**57**
**E139-K77**	**29**	E104-K94	26	E87-R90	34	E54-R74	60
D78-K75	43	E139-R74	63	D50-R74	25	E87-R90	22
**D80-K75**	**24**	**E139-K77**	**87**	D78-K75	73	E104-K94	41
D122-R106	53	D22-K21	23	**D80-K75**	**43**	**E139-K77**	**81**
		D78-K75	27	D122-R106	76	D22-K21	23
		D95-K94	28	D122-R126	29	D78-K75	67
		D122-R106	61			D80-K75	44
						D95-K94	42
						D122-R106	47

* Key salt-bridge occupancies for IV that change substantially across conditions are shown in bold.
